# Structural Exploration of Zirconium Metal–Organic Frameworks Through Linker Desymmetrization and Modulator Compensation

**DOI:** 10.1002/adma.202514373

**Published:** 2025-12-22

**Authors:** Rong‐Ran Liang, Kun‐Yu Wang, Zongsu Han, Kui Tan, Yihao Yang, Zhaoyi Liu, Joshua Rushlow, Jiatong Huo, Hong‐Cai Zhou

**Affiliations:** ^1^ Department of Chemistry Texas A&M University College Station Texas USA; ^2^ School of Energy and Environment Southeast University Nanjing Jiangsu China; ^3^ Department of Chemistry University of North Texas Denton Texas USA

**Keywords:** linker‐desymmetrization‐modulator‐compensation, metal–organic frameworks, ordered structural defects, selective gas adsorption

## Abstract

Zirconium‐based metal‐organic frameworks (Zr‐MOFs) feature exceptional thermal/chemical stability among various MOFs, enabling diverse applications. Their material properties are highly dependent on molecular structures consisting of Zr‐clusters and organic linkers. However, structural diversity in Zr‐MOFs remains constrained by the limited variety of known Zr‐clusters and the predominance of high‐symmetry linkers, which stems from the inherent symmetry constraints presented by Zr‐clusters. In this work, we develop a linker‐desymmetrization‐modulator‐compensation (LDMC) strategy to construct Zr‐MOFs with enhanced structural diversity. This approach reduces linker symmetry to create structural defects in Zr‐clusters, while such a thermodynamically unfavorable process can be compensated for by the coordination of modulators, such as benzoic acid and formic acid. As a result, two MOFs, PCN‐1005 and PCN‐1006, with unprecedented Zr‐clusters have been constructed. PCN‐1005 features an asymmetric Zr_6_ cluster stabilized by mono‐ and capping benzoates. In PCN‐1006, a rare pentacarboxylate linker enables the formation of a dual‐node network comprised of both Zr_6_ and Zr_6_‐f‐Zr_6_ clusters, resulting in one‐dimensional channels with exceptional adsorption performance for methane and carbon dioxide, affording high selectivity over hydrogen. These findings underscore the advancement of the LDMC strategy in promoting the structural complexity and functionality of Zr‐MOFs, providing a versatile platform for energy and environmental applications.

## Graphical abstract

A linker‐desymmetrization‐modulator‐compensation (LDMC) strategy for constructing structurally diverse Zr‐MOFs is reported. This approach reduces linker symmetry to create defects in Zr‐clusters, which can be compensated for by modulator coordination. Using this approach, we synthesized PCN‐1005 and PCN‐1006, featuring unprecedented Zr‐clusters. In PCN‐1006, a pentacarboxylate linker enables the formation of a dual‐node network, delivering exceptional CH_4_/CO_2_ adsorption and H_2_ selectivity.

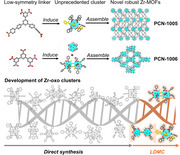

## 1 Introduction

**FIGURE 1:**
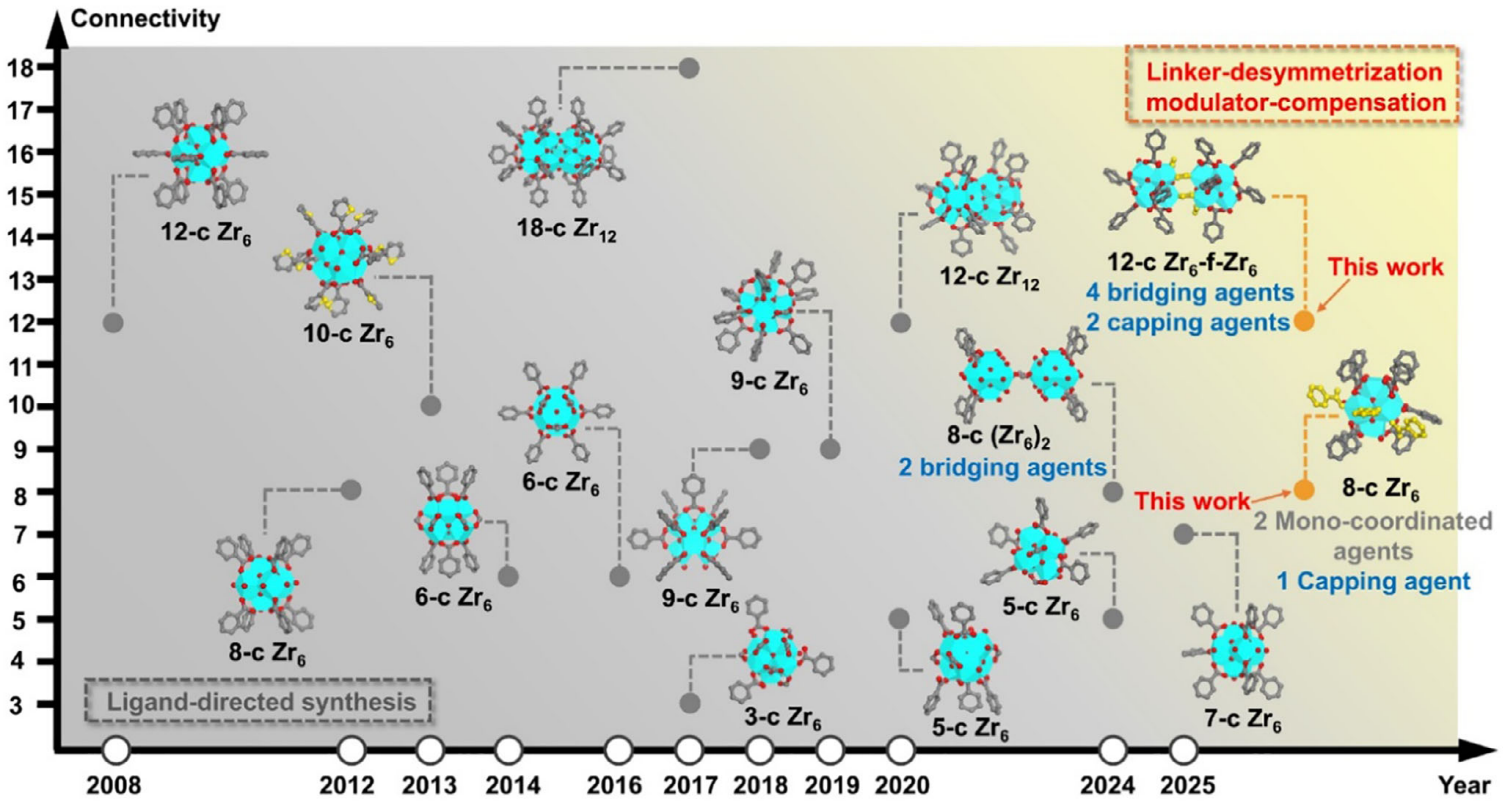
The chronicle of Zr‐clusters reported in MOFs and illustration of the two Zr‐clusters developed through the LDMC strategy. C, O, and Zr atoms are represented by gray, red, and cyan, respectively. Atoms in coordinated modulator ligands are highlighted in yellow. Hydrogen atoms in the structures are omitted for clarity.

Intricate interplay between structure and functionality is a defining characteristic of natural systems, where biological materials exhibit remarkable adaptability to fulfill diverse functional demands [1, 2, 3]. Such a profound relationship has inspired the development of materials science, where structural design serves as a cornerstone for discovering advanced materials with tailored functionalities. Among these, metal‐organic frameworks (MOFs) have emerged as materials with exceptional structural diversity and versatility. These porous crystalline materials, composed of inorganic metal clusters and organic linkers connected by coordination bonds, feature unparalleled tunability in terms of porosity, crystallinity, and functionality [4, 5, 6, 7]. Such properties have positioned MOFs as versatile platforms for applications, including gas storage and separation [8, 9], catalysis [10, 11], electronic devices [12, 13], sensing [13, 14], drug delivery [15, 16], and wastewater remediation [17, 18, 19]. Zirconium‐based MOFs (Zr‐MOFs) are engaging materials for practical applications due to their high stability [20, 21], which arises from strong Zr(IV)‐carboxylate coordination in line with Pearson's hard/soft acid/base (HSAB) principle [22, 23]. However, the structural exploration of Zr‐MOFs remains largely limited to frameworks built from highly symmetric ligands and conventional eight‐ or twelve‐connected Zr_6_ clusters [24, 25, 26], which constrain their topological diversity and limit their potential for innovative functions. Through the years, extensive efforts have been devoted to regulating synthetic conditions and linker design, leading to the discovery of various Zr‐clusters, including Zr_6_ and Zr_12_ clusters with varying connectivity (Figure 1), which are set as essential cornerstones in exploring the structural diversity of Zr‐MOFs [25, 27, 28, 29, 30, 31, 32, 33, 34, 35, 36, 37, 38, 39, 40]. Nevertheless, most reported Zr‐clusters have been discovered serendipitously. So far, there is a lack of effective strategies to controllably synthesize MOFs featuring Zr‐clusters with low symmetry and structural defects, which is thermodynamically unfavorable.

Recently, our group have sought to develop novel Zr‐MOFs through linker desymmetrization. This method reduces the symmetry of the linker, creating controlled structural defects that result in unprecedented metal clusters and intricate MOF structures. Such an advanced synthetic approach can be exemplified by the synthesis of PCN‐999, which showcases two distinct metal nodes: a Zr_6_ cluster and a bi‐formate‐bridged (Zr_6_)_2_ cluster. The formates act as bridging agents within the (Zr_6_)_2_ node, which compensates for the high energy of the structural defects and thus stabilizes the integral framework [18]. Similarly, Liu et al. reported the synthesis of Zr‐pentacarboxylate frameworks (HIAM‐4040 and HIAM‐4040‐OH) featuring unprecedented 5‐connected Zr_6_ clusters. In these frameworks, formates act as capping agents, compensating for reduced symmetry and stabilizing the overall structure [36]. Despite these advances, the systematic investigation of the principles underlying linker desymmetrization and defect compensation remains underexplored.

Herein, we introduce the linker‐desymmetrization‐modulator‐compensation (LDMC) strategy for engineering ordered defects and developing the novel MOF architectures. This approach combines desymmetrized linkers—which introduce mismatched coordination environments—with molecular modulators (e.g., benzoic acid, formic acid) that stabilize defective clusters via capping or bridging (Scheme S1), enabling the formation of periodically ordered frameworks. The versatility of the LDMC strategy is demonstrated through the synthesis of PCN‐1005 and PCN‐1006. In PCN‐1005, a desymmetrized tetratopic ligand directs the formation of an asymmetric Zr_6_ cluster stabilized by two mono‐coordinated benzoates and a capping benzoate, which leads to the assembly of a 3D framework with an unprecedented topology and irregular pores. PCN‐1006 employs a rare pentacarboxylic acid to generate two types of metal nodes: the typical Zr_6_ cluster and a novel Zr_6_‐f‐Zr_6_ cluster, in which two Zr_6_ clusters are bridged by four formates and capped by two additional formates. The interplay between the pentacarboxylate ligands and these Zr‐clusters produces a 3D network featuring 1D open channels aligned along the *b*‐axis. In both cases, modulators play a critical role in compensating for defects caused by reduced linker symmetry, ensuring the periodic arrangement of structural units. Furthermore, PCN‐1006 demonstrates outstanding adsorption capacities for methane and carbon dioxide, with high selectivity over hydrogen, attributed to the presence of multiple formates and a suitable pore size.

## 2 Results and Discussion

### 2.1 Synthesis and Structure Analysis of PCN‐1005

**FIGURE 2:**
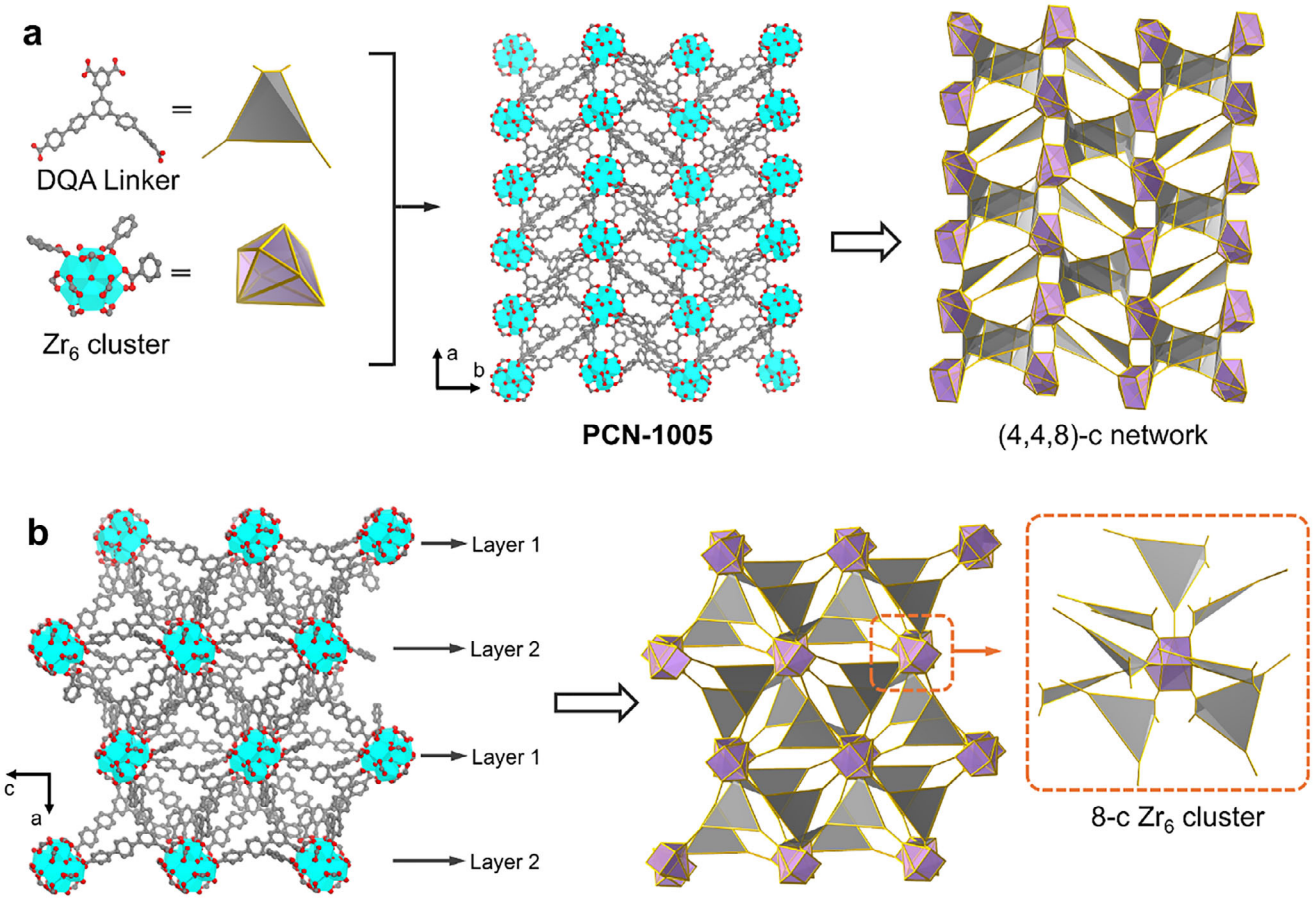
(a) Demonstration of the DQA linker and the A‐Zr_6_ cluster, along with the 3D structure of PCN‐1005 showing an unprecedented topology. (b) Demonstration of the connection between the DQA linker and the A‐Zr_6_ cluster in PCN‐1005. C, O, and Zr atoms are represented by gray, red, and cyan, respectively. Hydrogen atoms in the structures are omitted for clarity.

Inspired by the desymmetrized ligand design of PCN‐999, another tetratopic ligand with greater asymmetry in branch length (Figure S1), 5''‐(3,5‐dicarboxyphenyl)‐[1,1':4',1'':3'',1''':4''',1''''‐quinquephenyl]‐4,4''''‐dicarboxylic acid (H_4_DQA), was employed to synthesize the novel Zr‐MOF. The synthesis leveraged the LDMC strategy, facilitating the formation of the unique framework, PCN‐1005 (Figure 2a). PCN‐1005 was obtained via solvothermal reactions involving ZrCl_4_ and H_4_DQA in a mixture of dimethylformamide (DMF) and benzoic acid. Block‐shaped single crystals were isolated and determined via single‐crystal X‐ray diffraction (SCXRDo crystallize in the *P*2_1_2_1_2_1_ space group with lattice parameters *a* = 34.4104(3) Å, *b* = 25.3123(2) Å, *c* = 20.7123(2) Å, and *α* = *β* = *γ* = 90° (Table S1).

An asymmetric cluster, [Zr_6_(*µ*_3_‐O)_4_(*µ*_3_‐OH)_4_(OH)(H_2_O)_3_(C_6_H_5_COO)_3_(COO)_8_] (A‐Zr_6_), was generated during MOF construction, featuring two mono‐coordinated benzoates and a capping benzoate (Figure S2). Notably, the reduced symmetry of the DQA linker imposed an irregular coordination environment, resulting in defects within the A‐Zr_6_ cluster. These defects were simultaneously mitigated through compensation by benzoic acid modulators. Each asymmetric A‐Zr_6_ cluster is connected to eight DQA linkers in a non‐uniform arrangement (Figure 2b), while each DQA linker bridges four A‐Zr_6_ clusters, leading to a complex 3D framework with an unprecedented topology, which is a 3‐nodal 4,4,8‐c net with stoichiometry (4‐c)(4‐c)(8‐c) (Figure S3). The resulting structure contains interconnected pores of irregular shapes. The voids occupied by disordered guest solvent molecules like DMF within PCN‐1005 are calculated to account for ∼30% of the unit cell volumes (Figure S4).

### 2.2 Synthesis and Structure Analysis of PCN‐1006

**FIGURE 3:**
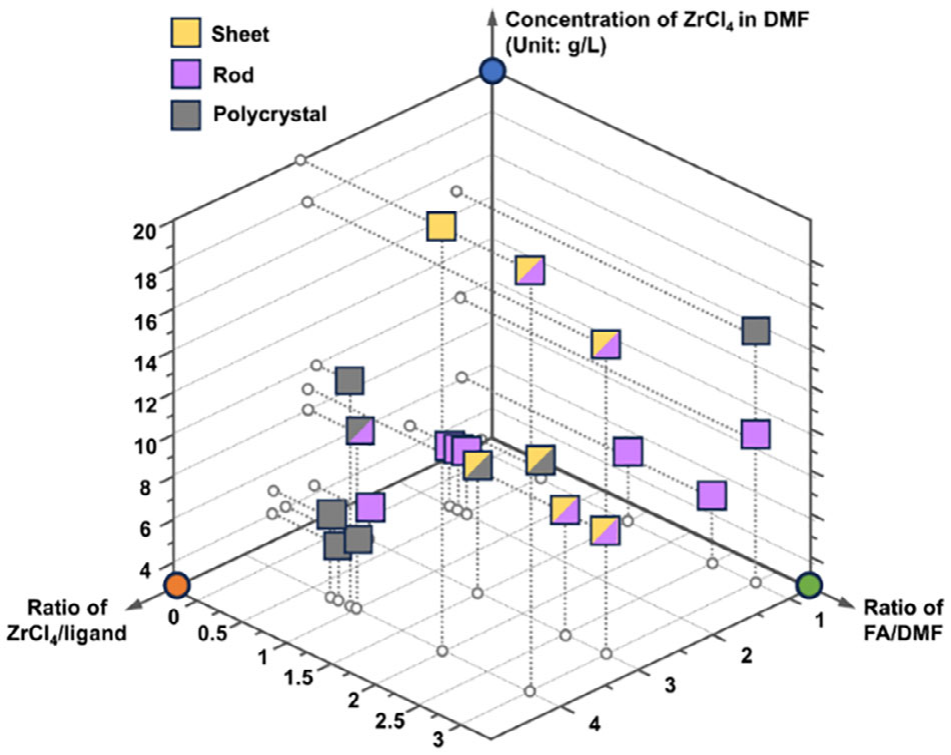
Crystal morphology regulation during PCN‐1006 synthesis.

The pentacarboxylic acid‐based ligand, [1,1':3',1''‐terphenyl]‐3,3'',5,5',5''‐pentacarboxylic acid (H_5_TPA), was designed through the linker desymmetrization strategy, which can be regarded as the derivatives from trimesic acid by elongating two arms and doubling their coordination sites (Figure S5). PCN‐1006 was synthesized through a solvothermal reaction using ZrCl_4_ and H_5_TPA in a mixture of DMF and formic acid. Our experimental results indicate that modulator choice is critical for crystallinity; for example, while formic acid successfully produced PCN‐1006, acids like acetic acid and trifluoroacetic acid inhibited crystallization, likely due to unsuitable acidity and steric effects. Interestingly, the crystal morphology of PCN‐1006 is highly dependent on synthetic conditions. Through a systematic investigation varying four parameters, namely amounts of metal salt, ligand, formic acid, and DMF, it was observed that higher concentrations of ZrCl_4_ tend to yield sheet‐shaped crystals, whereas rod‐shaped crystals are dominant at lower concentrations, based on the scanning electron microscopy (SEM) images (Figure S6). Such a result indicates that crystal morphology is highly associated with the initial nucleation stage of the MOF. Furthermore, increasing the formic acid concentration can enlarge crystal size, as the formic acid can act as a modulator to stabilize coordinatively unsaturated Zr‐clusters during the MOF growth (Figure 3). Therefore, the process of modulator‐induced stabilization likely involves a delicate entropic‐enthalpic balance, wherein selective modulator binding reduces the overall free energy of low‐symmetry configurations [41, 42].

**FIGURE 4:**
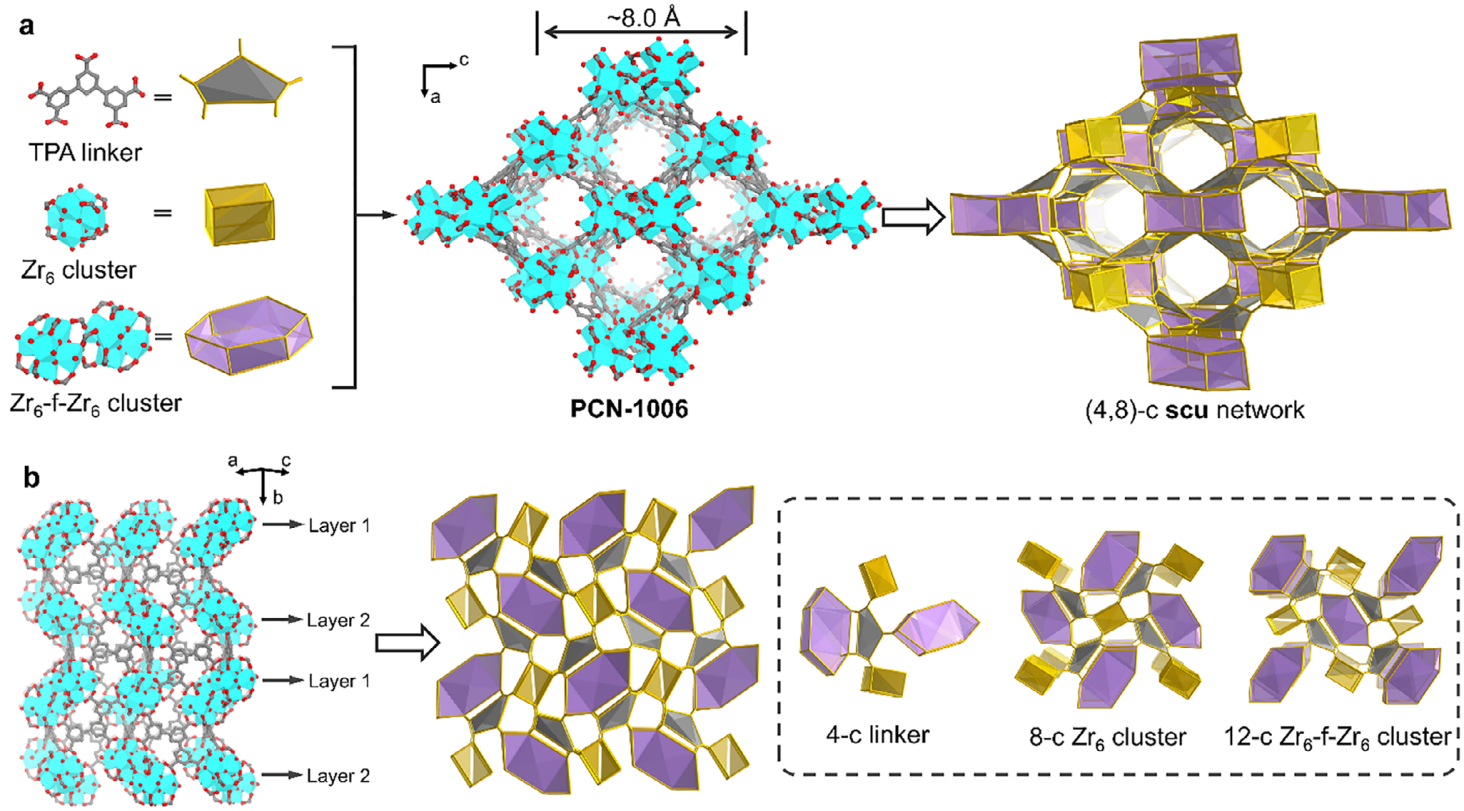
(a) Demonstration of TPA linker and the two types of clusters, along with the 3D structure of PCN‐1006 showing 1D channels along the *b*‐axis. (b) Demonstration of the connection between the TPA linker and the two clusters in PCN‐1006. C, O, and Zr atoms are represented by gray, red, and cyan, respectively. Hydrogen atoms in the structures are omitted for clarity.

SCXRD study revealed that PCN‐1006 crystallized in the *P*2_1_/*n* space group with lattice parameters *a* = 17.5286(15) Å, *b* = 27.933(2) Å, *c* = 22.6490(17) Å, *α* = *γ* = 90°, and *β* = 90.790(5)° (Table S1). Notably, two types of metal nodes are produced in the process of MOF synthesis, one is the typical [Zr_6_(*µ*_3_‐O)_4_(*µ*_3_‐OH)_4_(OH)_4_(H_2_O)_4_(COO)_8_] (Zr_6_) cluster and the other one is a unique {[Zr_6_(*µ*_3_‐O)_4_(*µ*_3_‐OH)_4_(OH)_3_(H_2_O)_3_(HCOO)(COO)_6_]_2_(HCOO)_4_} (Zr_6_‐f‐Zr_6_) cluster (Figure 4a). The Zr_6_‐f‐Zr_6_ cluster is composed of two Zr_6_ clusters bridged by four formates, where two additional capping formates are observed (Figure S7). Although a bi‐formate‐bridged (Zr_6_)_2_ cluster has been reported in the construction of PCN‐999, this is the first time that such a combination of two Zr_6_ clusters are assembled utilizing both bridging and capping formates to compensate for the defects generated by the linker desymmetrization in Zr‐MOFs.

While the Zr_6_ cluster connects to eight carboxylates from eight distinct ligands (Figure 4b), the Zr_6_‐f‐Zr_6_ node links with 12 carboxylates from eight ligands, where four ligands bridge the two Zr_6_ moieties in the Zr_6_‐f‐Zr_6_ node due to their unique penta‐geometry (Figure S8). As a result, each ligand connects to two Zr_6_ clusters and two Zr_6_‐f‐Zr_6_ nodes with one of the Zr_6_‐f‐Zr_6_ nodes coordinating with two carboxylates of the ligand simultaneously (Figure S9). These pentacarboxylates based linkers serve as four faces and four Zr_6_ clusters along with four Zr_6_‐f‐Zr_6_ nodes arranged alternatively to constitute eight vertices, leading to the formation of deformed cubic cages (Figure S10). These cages were arranged vertically along the *b*‐axis to yield a novel 3D network with **scu** topology (Figure S11). The Zr_6_ and Zr_6_‐f‐Zr_6_ clusters are arranged alternatively along the *b*‐axis, resulting in the formation of the 1D open channels with a rhombic geometry (∼8.0 Å). The voids occupied by disordered guest solvent molecules within PCN‐1006 are calculated to be ∼27% of the unit cell volume (Figure S12). The large void volume, along with 1D open channels and the unique Zr‐clusters, endows PCN‐1006 with promising potential for gas sorption and separation.

### 2.3 Porosity and Stability Analysis

Powder samples were prepared to further characterize the MOFs by slightly modifying the synthetic conditions. The crystalline structures of both MOFs in their powder form were verified by the powder X‐ray diffraction (PXRD) patterns, which reproduced the calculated patterns obtained from their single crystal data (Figure S13). SEM images reveal flower cluster‐like morphology for PCN‐1005 (Figure S14). Their thermal stability was evaluated by thermogravimetric analysis (TGA), which showed two‐step weight loss with the increase of the temperature. The first step occurred at ∼100°C for PCN‐1005 and ∼200°C for PCN‐1006, which should be attributed to the loss of the coordinated benzoates/formates, further validating the participation of the modulators in the construction of both MOFs. The second step corresponding to the decomposition of the MOF frameworks was as high as 400°C–500°C (Figure S15), suggesting high thermal stability of PCN‐1005 and PCN‐1006.

**FIGURE 5:**
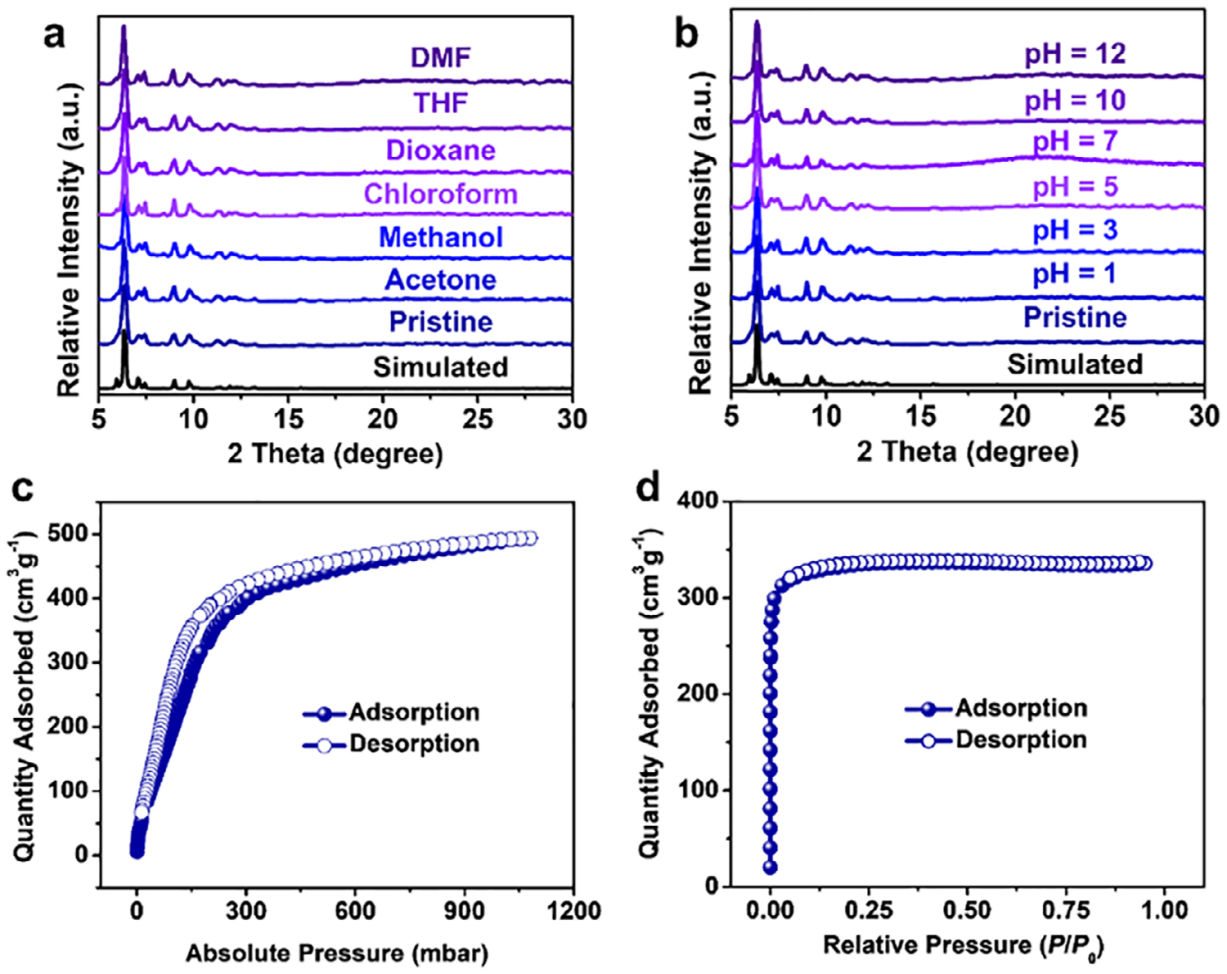
PXRD patterns of PCN‐1006 before and after exposure to (a) various organic solvents, and (b) aqueous solutions with different pH values. (c) CO_2_ adsorption‐desorption isotherm of PCN‐1005 at 195 K. (d) N_2_ adsorption‐desorption isotherm of PCN‐1006 at 77 K.

To further investigate the chemical stability of the Zr‐MOFs, we immersed PCN‐1005 and PCN‐1006 into different organic solvents and aqueous solutions and tested their crystallinity through PXRD. PCN‐1005 was found to show limited stability, with diffraction peaks weakening and broadening upon solvent immersion. Notably, its crystallinity is almost entirely lost in water (Figure S16). This is likely due to its A‐Zr_6_ clusters, where the desymmetrization strategy introduces localized strain and weaker coordination sites. These defects result in: (1) incomplete coordination or weaker Zr─O bonds, which make the cluster more vulnerable to solvent attack; and (2) higher thermodynamic instability. Although modulator compensation (e.g., benzoic acid) is used, the structure of the framework is not robust enough to withstand aggressive solvents. Consequently, the instability of PCN‐1005 under these conditions limits its application in areas requiring prolonged liquid‐phase exposure. In contrast, PCN‐1006 was found to retain its crystalline structure upon treatment with various solvents, including DMF, *p*‐dioxane, tetrahydrofuran (THF), methanol, chloroform, and acetone (Figure 5a). Furthermore, the PXRD patterns remained identical before and after immersing PCN‐1006 into aqueous solutions with a wide range of pH values (1 to 12) (Figure 5b), highlighting its remarkable chemical stability. This robustness originates from its dual‐node network of symmetric Zr_6_ and Zr_6_‐f‐Zr_6_ clusters connected by a pentacarboxylate linker. This highly connected and symmetrical structure distributes structural stress more evenly and achieves improved thermodynamic stability, as the modulator compensation (e.g., formic acid) effectively stabilizes the clusters during synthesis, reducing structural defects.

The porosity of PCN‐1005 was characterized using CO_2_ adsorption‐desorption isotherm measured at 195 K. A sharp increase in the adsorption curve at low pressure (< 0.2 bar) confirmed the presence of permanent micropores (Figure 5c). The observed desorption hysteresis can be attributed to the capillary condensation in the micropores [43, 44]. The maximum CO_2_ uptake reached 494 cm^3^g^−1^ at ∼1.0 bar. Analysis of the adsorption isotherm yielded Brunauer–Emmett–Teller (BET) surface area of 622 m^2^g^−1^ (Figure S17). The total pore volume of PCN‐1005 was determined to be 0.74 cm^3^g^−1^ (at *P/P*_0_ = 0.90). However, nitrogen adsorption at 77 K showed negligible uptake (Figure S17), consistent with the narrow pore size of the material. In comparison, the porosity of PCN‐1006 was assessed through nitrogen adsorption‐desorption isotherm at 77 K. A steep increase was also observed in the adsorption curve at very low relative pressure (< 0.01 *P*/*P*_0_), indicating the presence of permanent micropores (Figure 5d). The BET surface area of PCN‐1006 were calculated to be 1315 m^2^g^−1^ (Figure S18). The total pore volume was determined to be 0.52 cm^3^g^−1^ (at *P*/*P*_0_ = 0.95). The pore size distribution of PCN‐1006 was determined using a slit‐pore, carbon‐based density functional theory (DFT) model, which showed a main distribution at ∼8.0 Å (Figure S18), matching well with the theoretical pore dimension calculated in its single crystal data.

### 2.4 PCN‐1006 for Hydrogen Purification

Motivated by the unique Zr_6_‐f‐Zr_6_ node and the microporous architecture of PCN‐1006, its performance in gas adsorption and separation was systematically explored. In recent years, there has been a surge of global interest in hydrogen as a clean energy carrier. Beyond its energy applications, hydrogen is essential for hydrocracking and hydrotreating processes in refinery units [45]. Currently, over 90% of global commercial hydrogen production relies on fossil fuels, such as methane or coal, with steam methane reforming (SMR) being one of the principal industrial methods [46]. This form of hydrogen, commonly called “gray” hydrogen [47], often contains impurities like CH_4_ and CO_2_, necessitating further purification to meet the stringent purity requirements of downstream applications.

**FIGURE 6:**
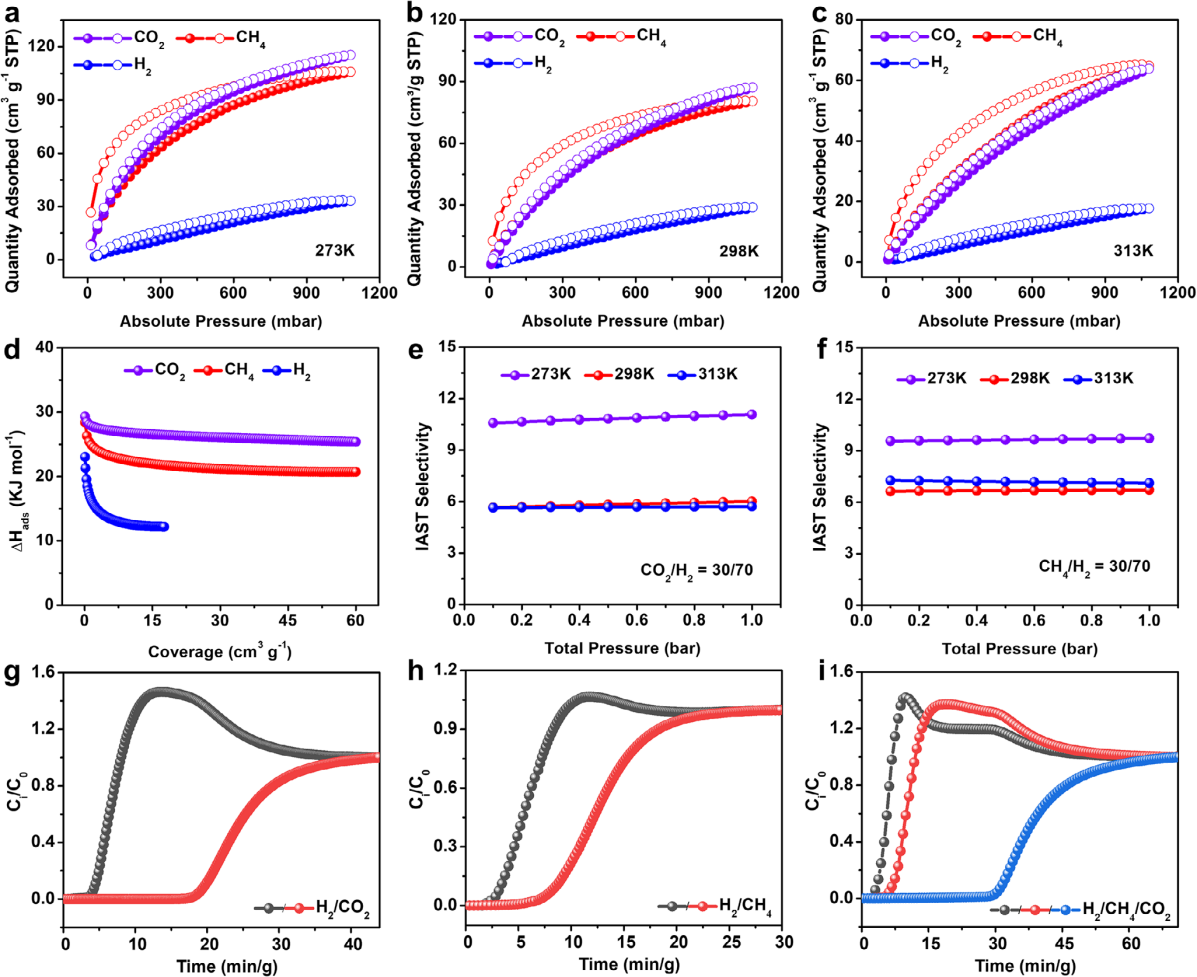
CO_2_, CH_4_, and H_2_ adsorption‐desorption isotherms of PCN‐1006 record at (a) 273 K, (b) 298 K, and (c) 313 K. (d) Isosteric heat of adsorption on PCN‐1006 for CO_2_, CH_4_, and H_2_, averaged over temperatures 273, 298, and 313 K. IAST selectivity of (e) CO_2_/H_2_ (30/70) and (f) CH_4_/H_2_ (30/70). Breakthrough curves for (g) CO_2_/H_2_ (30/70), (h) CH_4_/H_2_ (30/70), and (i) CO_2_/CH_4_/H_2_ (15/15/70) obtained over PCN‐1006 at 298 K and 1 bar.

To evaluate its potential for hydrogen purification, the pure gas adsorption‐desorption isotherms of PCN‐1006 for CH_4_, CO_2_, and H_2_ were measured at 273, 298, and 313 K under pressures up to 1 bar (Figure 6a–c). A fast increase in CH_4_ and CO_2_ uptake was observed at low pressure (< 0.5 bar), followed by a steady growth at higher pressures across all temperatures. Conversely, the H_2_ adsorption isotherms exhibited a continuous, gradual increase without signs of saturation over the entire pressure range. At 1 bar, PCN‐1006 exhibited high CO_2_ adsorption capacities of 115, 87, and 64 cm^3^g^−1^ at 273, 298, and 313 K, respectively. Similarly, the CH_4_ uptake capacities were 106, 81, and 64 cm^3^g^−1^ under the same conditions. In contrast, H_2_ uptake was significantly lower, with values of 33, 29, and 18 cm^3^g^−1^ at 273, 298, and 313 K, respectively. These findings indicate a strong affinity of PCN‐1006 for CH_4_ and CO_2_, likely due to the synergistic effects of its unique Zr_6_‐f‐Zr_6_ node and well‐matched pore dimensions. The observed hysteresis for CH_4_ can be attributed to adsorption‐induced structural deformation of the porous material, which alters pore throat size and induces different energies for gas adsorption and desorption [44, 48].

The isosteric heats of adsorption (ΔH_ads_) provided further insight into the adsorption behavior (Figure S19–S21). For CO_2_, the heat of adsorption decreased from 29.3 to 25.4 kJ mol^−1^ with increasing coverage, reflecting physisorption dominated by the progressive covering of the most thermodynamically favorable adsorption sites, followed by less favorable sites. A similar trend was observed for CH_4_, with the heat of adsorption decreasing from 28.4 to 20.7 kJ mol^−1^. In the case of H_2_, the heat of adsorption was markedly lower, reaching 12.0 kJ mol^−1^, indicating the weaker interaction of H_2_ with the framework compared to CH_4_ and CO_2_ (Figure 6d). These findings suggest the potential of PCN‐1006 for hydrogen purification from CH_4_ and CO_2_ impurities.

To assess the selectivity of PCN‐1006 for gas separation, Ideal Adsorption Solution Theory (IAST) selectivity calculations were performed for CO_2_/H_2_ and CH_4_/H_2_ mixtures at compositions of 30/70, 10/90, and 1/99, across all three temperatures. The selectivity for all mixtures remained nearly constant as pressure increased. At 1 bar, the selectivity for CO_2_/H_2_ (30/70) was 11.1, 6.0, and 5.7 at 273, 298, and 313 K (Figure 6e), respectively, while the selectivity for CH_4_/H_2_ (30/70) was 9.7, 6.7, and 7.1 under the same conditions (Figure 6f). Moreover, it is observed that selectivity remains largely unaffected by changes in gas composition (Figure S22). This behavior is consistent with the significantly stronger adsorption affinities of CO_2_ and CH_4_ compared to H_2_, which dominate the adsorption process even at low concentrations. The best separation performance was observed at the lowest temperature (273 K) for all binary mixtures. PCN‐1006 demonstrates moderate performance compared to established materials in the field. Its CH_4_/H_2_ separation is comparable to that of MOF‐74 variants, while its CO_2_/H_2_ selectivity is on par with MOF‐5, MOF‐177, BeBTB, and CoBDP (Table S2) [49, 50, 51, 52, 53, 54, 55]. These results suggest that PCN‐1006 is a highly promising candidate for CO_2_/H_2_ and CH_4_/H_2_ separation. This remarkable performance can be attributed to the optimized pore size and pore environment achieved through the LDMC strategy.

In addition, we conducted column breakthrough experiments using gas mixtures of CO_2_/H_2_ (30/70), CH_4_/H_2_ (30/70), and CO_2_/CH_4_/H_2_ (15/15/70) at ambient temperature and pressure to evaluate the dynamic separation performance of PCN‐1006. The CO_2_/H_2_ breakthrough curve shows that H_2_ breaks through at ∼4 min (at 5% of inlet concentration), while CO_2_ is retained for about 19 min (Figure 6g), indicating strong selectivity toward CO_2_ over H_2_. In the CH_4_/H_2_ experiment, H_2_ breaks through within 3 min, whereas CH_4_ is retained for around 8 min (Figure 6h). Although the single‐component adsorption capacities for CH_4_ and CO_2_ are similar—reflecting comparable thermodynamic uptake—their markedly different breakthrough times arise from differences in diffusion kinetics and competitive adsorption, consistent with the fact that breakthrough profiles are influenced by both thermodynamic and kinetic factors [56]. Furthermore, the ternary CO_2_/CH_4_/H_2_ (15/15/70) experiment confirms these trends: H_2_ breaks through first (∼3 min), followed by CH_4_ (∼6 min) and CO_2_ (∼30 min) (Figure 6i). These results surpass those of established MOF‐74 variants, including MOF‐74(Ni) monoliths, AX‐Mg‐MOF‐74, and zeolite‐5A@MOF‐74 composites,[49, 50, 51] demonstrating the practical potential of PCN‐1006 for hydrogen purification applications. Following H_2_ purification, the adsorbed CO_2_ and CH_4_ were desorbed from PCN‐1006 through thermal regeneration at 120 °C under vacuum. The material maintained its separation performance over two consecutive breakthrough cycles with no significant decrease (Figure S23), confirming its recyclability for repeated H_2_ purification. To evaluate the structural stability of PCN‐1006 after H_2_ purification, PXRD measurement was performed on the material recovered from the breakthrough tests. The PXRD pattern matches well with that of the as‐synthesized sample (Figure S24), confirming that the framework maintains its crystallinity and structural integrity throughout the separation process.

### 2.5 In Situ Infrared Spectroscopy

**FIGURE 7:**
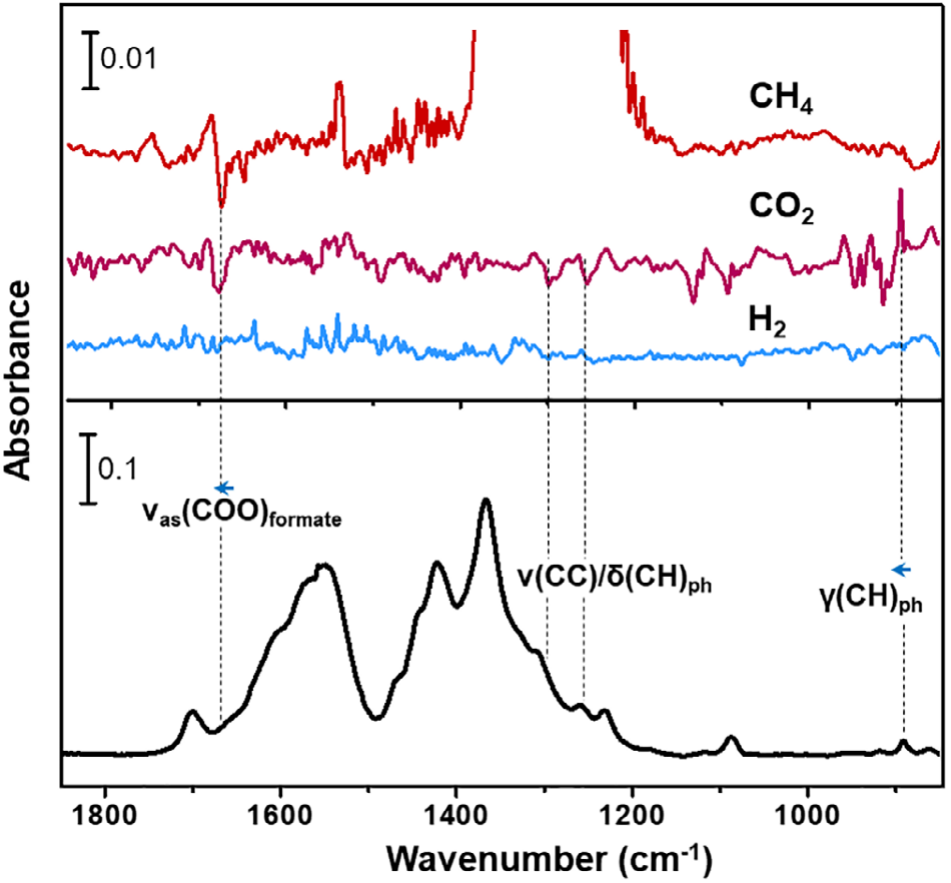
In situ IR spectra of loading CH_4_, CO_2_, and H_2_ into PCN‐1006 at 1 bar and 298 K. The Top panel shows the difference spectra obtained by referencing the spectra of the gas‐loaded MOF sample to that of the activated (pristine) one. The out‐of‐scale regions are due to the strong gas phase absorption. The bottom panel shows the spectrum of the activated MOF sample, referenced to blank KBr power. Notation and acronym: ν = stretching, δ = in‐plane deformation, γ = out‐of‐plane deformation, as = asymmetric, and ph = phenyl.

To investigate the interaction mechanism of CH_4_, CO_2_, and H_2_ within PCN‐1006, in situ infrared spectroscopy measurement was conducted on the sample upon loading CH_4_, CO_2_, and H_2_. Note that the gas phase signal is prohibitively strong for CO_2_ and CH_4_. We therefore focus our analysis on the perturbations occurring to the MOF modes in the difference spectra (Figure 7). The ν_as_(COO) mode of formate [57], is considerably perturbed upon CH_4_ and CO_2_ loading, typified by a noticeable blue‐shift (∼3 cm^−1^), indicating that the formate group interacts with CH_4_ and CO_2_ but not with H_2_. This observation agrees with the experimental gas adsorption results, confirming the functionality of the unique Zr_6_‐f‐Zr_6_ node enabled by the LDMC strategy. In addition, the γ(CH) mode of the middle phenyl ring on the organic linker at 891 cm^−1^ is appreciably blue‐shifted in position and enhanced in intensity. We thus infer that CO_2_ also interacts with this part, most likely in close proximity to the plane of the phenyl ring, which is further evidenced by the perturbation occurring to the phenyl modes ν(CC)/δ(CH) [58], as indicated by derivative‐like features in the difference spectra. In such a state, the presence of CO_2_ could produce an impediment to the out‐of‐plane CH deformation vibration [59], thereby steeping its potential and raising its frequency as observed in the spectrum (Figure 7).

## 3 Conclusion

In summary, we demonstrated the design of novel Zr‐MOFs by introducing the LDMC strategy, enabling precisely engineered structural complexity and functionality. The LDMC approach combines desymmetrized linkers and modulator‐mediated defect compensation to stabilize low‐symmetric metal clusters, thus establishing a systematic strategy for the controlled synthesis of Zr‐MOFs with ordered structural defects and advanced functionality. The success of this methodology is demonstrated through the synthesis of PCN‐1005 and PCN‐1006. PCN‐1005 features an asymmetric Zr_6_ cluster, stabilized by mono‐ and capping benzoates, resulting in a 3D framework with an unprecedented topology. PCN‐1006 incorporates a dual‐node system with both Zr_6_ and Zr_6_‐f‐Zr_6_ clusters, bridged and capped by formates, creating 1D open channels. Remarkably, PCN‐1006 exhibits exceptional gas adsorption properties, including high methane and carbon dioxide adsorption capacities with superior selectivity over hydrogen, which can be attributed to the presence of multiple formates compensating for the structural defects introduced by the desymmetrized linker. By enriching the fundamental toolkits of MOF synthesis, the LDMC strategy addresses critical limitations in Zr‐MOF design, especially the inherent symmetrical restrictions of Zr‐clusters, and paves the way for creating next‐generation materials for tackling grand challenges in energy storage and gas separation.

## 4 Experimental Section/Methods

### 4.1 Synthesis of PCN‐1005

H_4_DQA (5 mg), ZrCl_4_ (12 mg), benzoic acid (400 mg), and DMF (2 mL) were charged in a 4 mL Pyrex vial. After sonication of around 10 min, the mixture was then heated at 120°C for 5 days. After cooling to room temperature, the single crystals were harvested. The powder sample was obtained by mixing H_4_DQA (20 mg), ZrCl_4_ (48 mg), benzoic acid (800 mg), and DMF (4 mL) in a 20 mL vial, followed by heating at 120°C for 3 days to give the white powder (yield: ∼65%).

### 4.2 Synthesis of PCN‐1006

H_5_TPA (5 mg), ZrCl_4_ (20 mg), formic acid (2.5 mL), and DMF (2.5 mL) were charged in a 20 mL Pyrex vial. After sonication of around 10 min, the mixture was then heated at 120°C for 5 days. After cooling to room temperature, the single crystals were harvested. The powder sample was obtained by mixing H_5_TPA (20 mg), ZrCl_4_ (40 mg), formic acid (400 µL), and DMF (2 mL) in a 20 mL vial, followed by heating at 120°C for 3 days to give the white powder (yield: ∼76%).

### 4.3 Gas Sorption Measurement

N_2_/CH_4_/CO_2_/H_2_ adsorption‐desorption measurements were performed on a Micromeritics ASAP 2020 system. Prior to the measurements, the as‐synthesized MOFs were washed with DMF to remove the unreacted starting materials, followed by the exchange with acetone several times to remove the non‐volatile DMF. The resulting samples were then activated under vacuum at 120°C for 12 h. The N_2_ adsorption‐desorption isotherms were then collected at 77 K, from which the specific surface area was generated using the BET model. CH_4_/CO_2_/H_2_ adsorption‐desorption isotherms were collected at different temperatures.

### 4.4 Breakthrough Experiment

The activated sample (0.7261 g) was packed into a stainless‐steel column with an inner diameter of 4 mm and a length of 105 mm. The outlet effluent from the column was continuously monitored by a mass spectrometer (Hidden QGA quantitative gas analysis S3 system). The column packed with activated sample was first purged with a helium gas flow (8 mL/min) for 12 h at 120°C. During the breakthrough process, the gas mixture flow rate was 2 mL/min. The breakthrough experiments were conducted at 298 K and 1 bar.

### 4.5 In Situ Infrared Spectroscopy

In situ IR measurements were performed on a Nicolet is50 FTIR spectrometer using a liquid N_2_‐cooled mercury cadmium telluride (MCT‐A) detector. The spectrometer is equipped with a vacuum cell that is placed in the main compartment with the sample at the focal point of the infrared beam. The sample (∼2 mg) was gently pressed onto a KBr pellet and placed into a cell that is connected to a vacuum line for evacuation. The sample was activated by evacuation at 150°C for 3 h, and then cooled back to room temperature for CO_2_, CH_4_, and H_2_ adsorption measurements.

[CCDC 2452802 and 2452803 contain the supplementary crystallographic data for this paper. These data can be obtained free of charge from The Cambridge Crystallographic Data Centre via www.ccdc.cam.ac.uk/data_request/cif.]

## Conflicts of Interest

The authors declare no conflicts of interest.

## Supporting information




**Supporting File 1**: adma71841‐sup‐0001‐SuppMat.docx

**Supporting File 2**: adma71841‐sup‐0002‐Data.zip

## Data Availability

The data that support the findings of this study are available in the supplementary material of this article.
