# Supplementary material for: Structural Exploration of Zirconium Metal–Organic Frameworks Through Linker Desymmetrization and Modulator Compensation
Source: Adv Mater. 2025 Dec 22;38(10):e14373. doi: 10.1002/adma.202514373 (PMC12910544; doi:10.1002/adma.202514373)
Supplement: Supplementary file 1 — Supporting File 1: adma71841‐sup‐0001‐SuppMat.docx [file ADMA-38-e14373-s002.docx]

Supporting Information

**Structural Exploration of Zirconium Metal-Organic Frameworks through Linker Desymmetrization and Modulator Compensation**

Rong-Ran Liang, Kun-Yu Wang, Zongsu Han, Kui Tan, Yihao Yang, Zhaoyi Liu, Joshua Rushlow, Jiatong Huo, and Hong-Cai Zhou*

Contents

[Section 1. General methods and instruments 4](#_Toc208422405)

[Section 2. Characterizations of the MOFs 5](#_Toc208422406)

[Scheme S1. Schematic demonstration of the linker-desymmetrization-modulator-compensation strategy. 5](#_Toc208422407)

[Figure S1. Chemical structure of H_4_DQA. 5](#_Toc208422408)

[Figure S2. Demonstration of linker DQA, and the asymmetric Zr_6_ cluster with two mono-coordinated benzoates and a capping benzoate in PCN-1005 5](#_Toc208422409)

[Figure S3. Packing structure of PCN-1005 6](#_Toc208422410)

[Figure S4. The solvent accessible void of PCN-1005 6](#_Toc208422411)

[Figure S5. Design of the desymmetrized ligand H_5_TPA through the derivation from trimesic acid by elongating two arms and doubling their linking sites. 6](#_Toc208422412)

[Figure S6. SEM images of PCN-1006. 7](#_Toc208422413)

[Figure S7. Demonstration of Zr_6_, and Zr_6_-f- Zr_6_ clusters in PCN-1006. 7](#_Toc208422414)

[Figure S8. Demonstration of the coordination of the ligand with Zr_6_, and Zr_6_-f- Zr_6_ clusters in PCN-1006 7](#_Toc208422415)

[Figure S9. Demonstration of the connection of each ligand to the Zr_6_ and Zr_6_-f-Zr_6_ nodes in PCN-1006 8](#_Toc208422416)

[Figure S10. Demonstration of the deformed cubic cages within PCN-1006. 8](#_Toc208422417)

[Figure S11. Packing structure of PCN-1006 8](#_Toc208422418)

[Figure S12. Demonstration of the solvent accessible void of PCN-1006 9](#_Toc208422419)

[Figure S13. PXRD patterns of PCN-1005 and PCN-1006. 9](#_Toc208422420)

[Figure S14. SEM images of PCN-1005. 9](#_Toc208422421)

[Figure S15. TGA profiles of PCN-1005 and PCN-1006. 10](#_Toc208422422)

[Figure S16. PXRD patterns of PCN-1005 before and after exposure to organic solvents 10](#_Toc208422423)

[Figure S17. BET surface area plot and N_2_ adsorption-desorption isotherm of PCN-1005 10](#_Toc208422424)

[Figure S18. BET surface area plot and pore size distribution profile of PCN-1006. 11](#_Toc208422425)

[Figure S19. Langmuir Fit of CH_4_ uptake of PCN-1006 at different temperatures. 11](#_Toc208422426)

[Figure S20. Langmuir Fit of CO_2_ uptake of PCN-1006 at different temperatures. 11](#_Toc208422427)

[Figure S21. Langmuir Fit of H_2_ uptake of PCN-1006 at different temperatures. 12](#_Toc208422428)

[Figure S22. IAST selectivity of CO_2_/H_2_, CH_4_/H_2_, and CO_2_/CH_4_/H_2_ 12](#_Toc208422429)

[Figure S23. Breakthrough curves of different cycles for CO_2_/CH_4_/H_2_ over PCN-1006 12](#_Toc208422430)

[Figure S24. PXRD patterns of PCN-1006 before and after breakthrough cycles. 13](#_Toc208422431)

[Table S1. Crystallographic data and structural refinement summary. 13](#_Toc208422432)

[Table S2. Summary of gas sorption and separation performance of reported adsorbents. 14](#_Toc208422433)

[Reference 15](#_Toc208422434)

# Section 1. General methods and instruments

**Powder X-ray diffraction (PXRD).** PXRD patterns were collected on a Bruker D8 Advance ECO powder diffractometer with a Cu microfocus tube (*λ* = 1.54178 Å) at 40 kV and 25 mA.

**Thermogravimetric analysis (TGA).** TGA measurement was conducted on a Mettler-Toledo TGA/DSC 1 under nitrogen (N_2_) atmosphere with a ramp rate of 5 °C/min from room temperature and 800 °C.

**Scanning electron microscopy (SEM).** SEM was carried out using a FEI QUANTA 600 FE-SEM scanning electron microscope. The samples were dispersed over the slices of silicon wafer adhered to flat copper platform sample holders and then coated with gold using a sputter coater (ambient temperature, 85 torr pressure in a nitrogen atmosphere, puttered for 30s from a solid gold target at a current of 30 mA) before being submitted to SEM characterization.

**Single-crystal X-ray crystallography.** The single crystals of PCN-1005 and PCN-1006 were directly transferred from the mother liquid to the oil, and then mounted onto a loop for single crystal X-ray diffraction measurements (SCXRD). The data were collected either through synchrotron or a Synergy Rigaku Duo Mo/Cu micro-source HPC Kappa X-ray diffractometer. The data were collected using Cu X-ray radiation at 100K. The single crystal structures were solved and refined using Olex2 software.^[1]^ All structures were solved by the direct method using the *SHELXT* program and refined by full-matrix least-squares method with *SHELXL* package.^[2]^ All non-hydrogen atoms were refined with anisotropic displacement parameters, and the hydrogen atoms were positioned by geometrical calculation and then refined by riding. The free solvent molecules are highly disordered in MOFs and attempts to locate and refine the solvent peaks were unsuccessful. The diffused electron densities resulting from these solvent molecules were removed using the solvent MASK. Crystal data are summarized in Table S1 and the single crystal structures can be obtained free of charge from The Cambridge Crystallographic Data Centre with the CCDC numbers of 2452803 and 2452802.

# Section 2. Characterizations of the MOFs


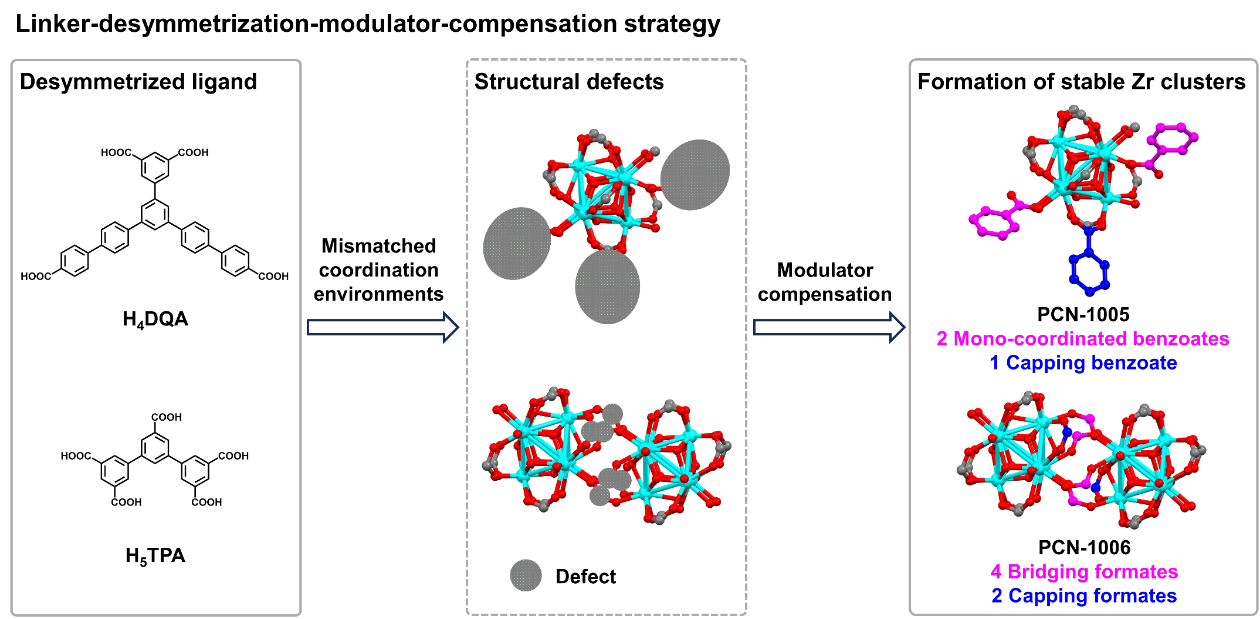


## Scheme S1. Schematic demonstration of the linker-desymmetrization-modulator-compensation strategy.


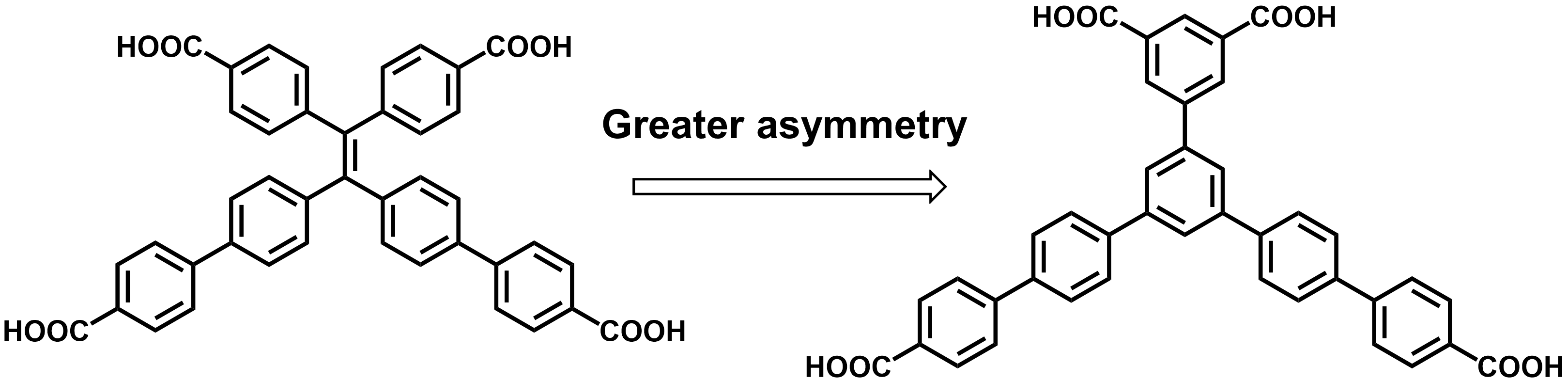


## Figure S1. Chemical structure of H_4_DQA.


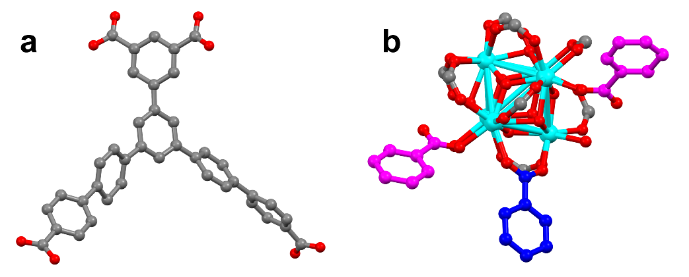


## Figure S2. Demonstration of (a) linker DQA, and (b) the asymmetric Zr_6_ cluster with two mono-coordinated benzoates and a capping benzoate in PCN-1005.


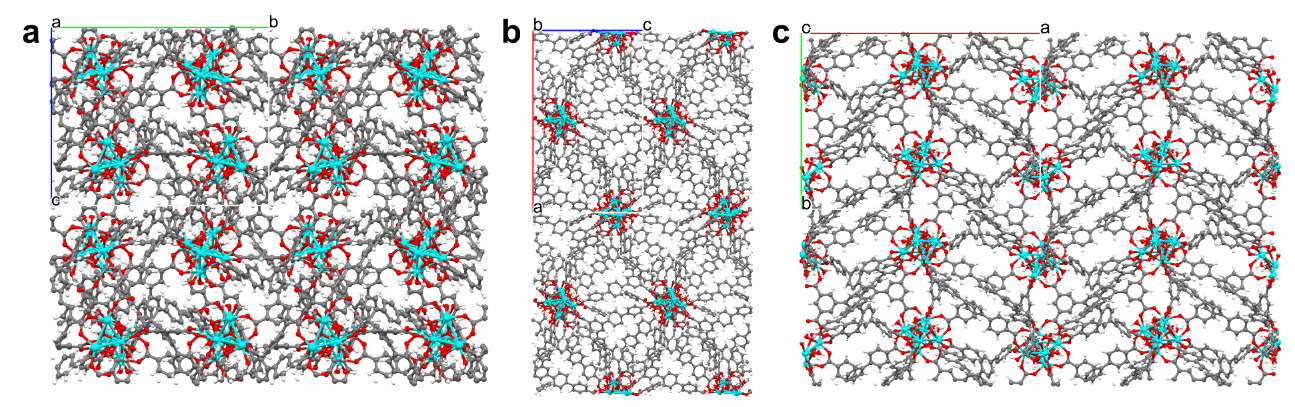


## Figure S3. Packing structure of PCN-1005 along (a) *a* axis, (b) *b* axis, and (c) *c* axis. C, O, and Zr atoms are represented by grey, red, and cyan, respectively. Hydrogen atoms are omitted for clarity.


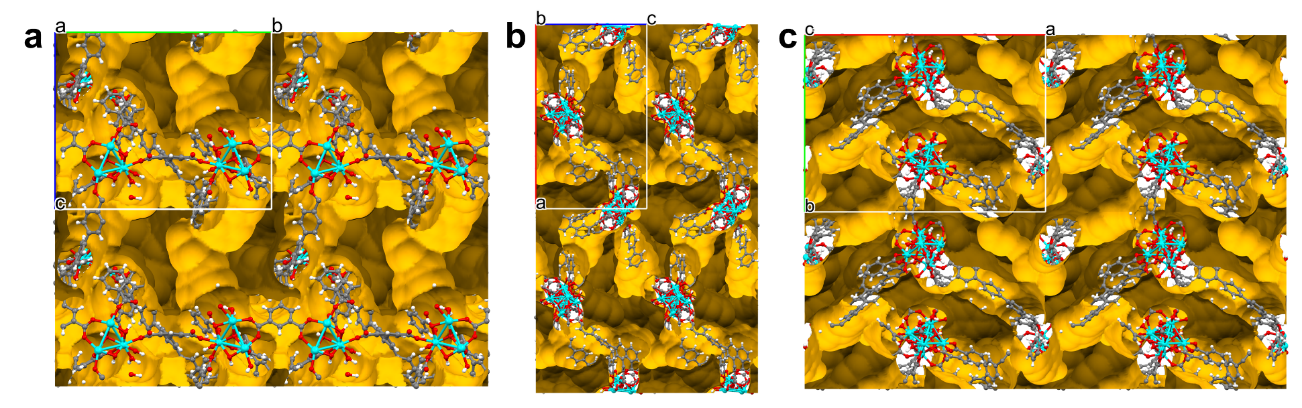


## Figure S4. The solvent accessible void of PCN-1005 along (a) *a* axis, (b) *b* axis, and (c) *c* axis. C, O, and Zr atoms are represented by grey, red, and cyan, respectively. Hydrogen atoms are omitted for clarity.


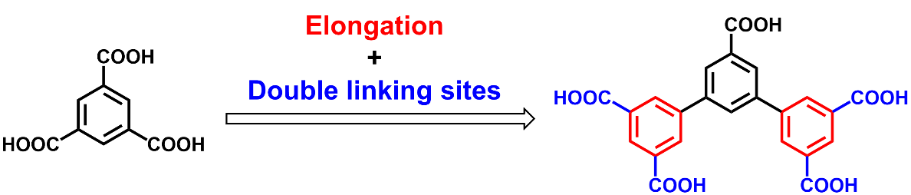


## Figure S5. Design of the desymmetrized ligand H_5_TPA through the derivation from trimesic acid by elongating two arms and doubling their linking sites.


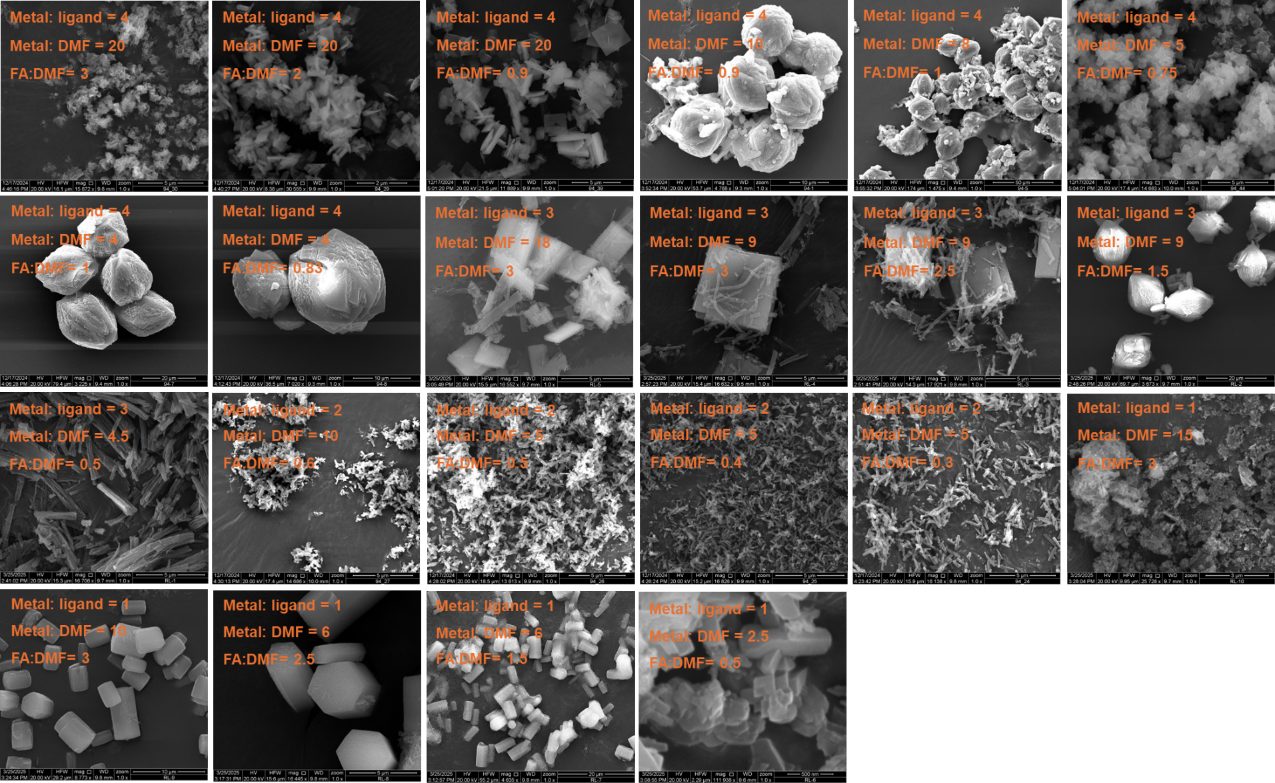


## Figure S6. SEM images of PCN-1006.


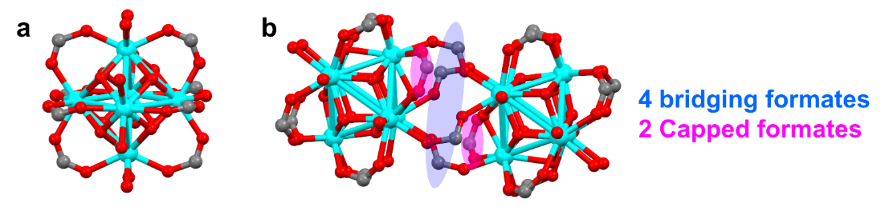


## Figure S7. Demonstration of (a) Zr_6_, and (b) Zr_6_-f- Zr_6_ clusters in PCN-1006. C, O, and Zr atoms are represented by grey, red, and cyan, respectively. Hydrogen atoms are omitted for clarity.


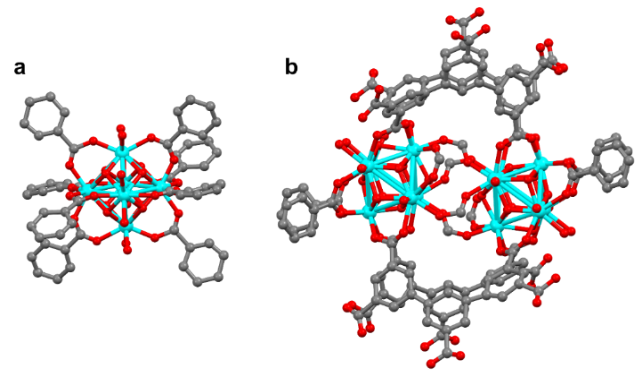


## Figure S8. Demonstration of the coordination of the ligand with (a) Zr_6_, and (b) Zr_6_-f- Zr_6_ clusters in PCN-1006. C, O, and Zr atoms are represented by grey, red, and cyan, respectively. Hydrogen atoms are omitted for clarity.


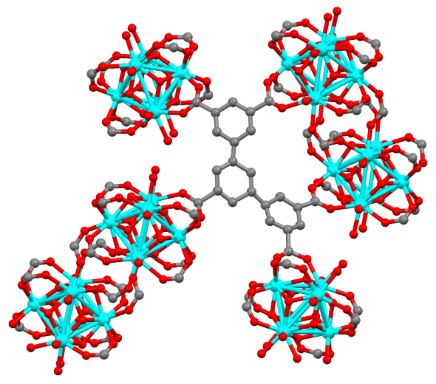


## Figure S9. Demonstration of the connection of each ligand to the Zr_6_ and Zr_6_-f-Zr_6_ nodes in PCN-1006. C, O, and Zr atoms are represented by grey, red, and cyan, respectively. Hydrogen atoms are omitted for clarity.


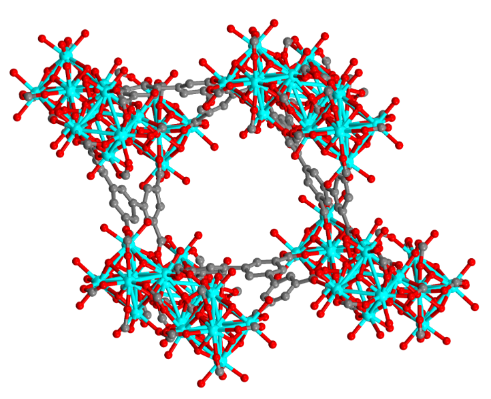


## Figure S10. Demonstration of the deformed cubic cages within PCN-1006. C, O, and Zr atoms are represented by grey, red, and cyan, respectively. Hydrogen atoms are omitted for clarity.


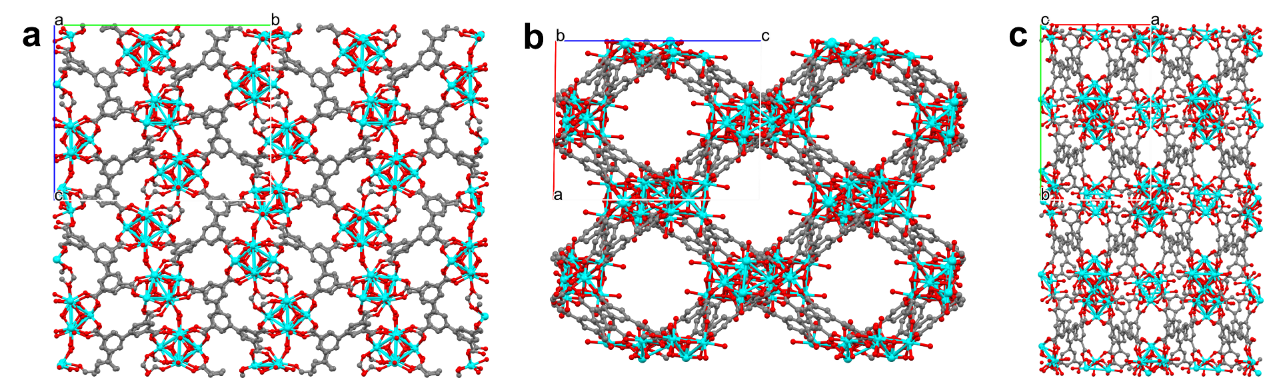


## Figure S11. Packing structure of PCN-1006 along (a) *a* axis, (b) *b* axis, and (c) *c* axis. C, O, and Zr atoms are represented by grey, red, and cyan, respectively. Hydrogen atoms are omitted for clarity.


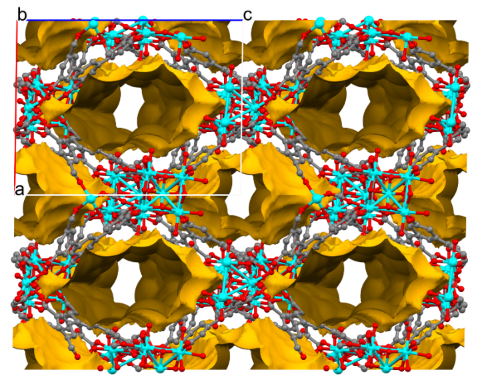


## Figure S12. Demonstration of the solvent accessible void of PCN-1006. C, O, and Zr atoms are represented by grey, red, and cyan, respectively. Hydrogen atoms are omitted for clarity.


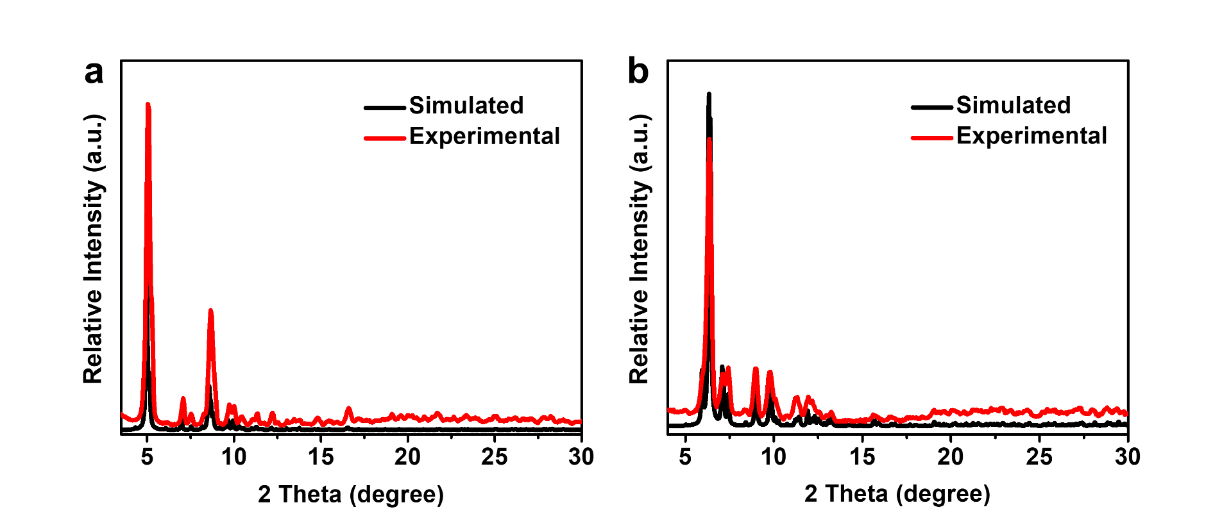


## Figure S13. PXRD patterns of (a) PCN-1005 and (b) PCN-1006.


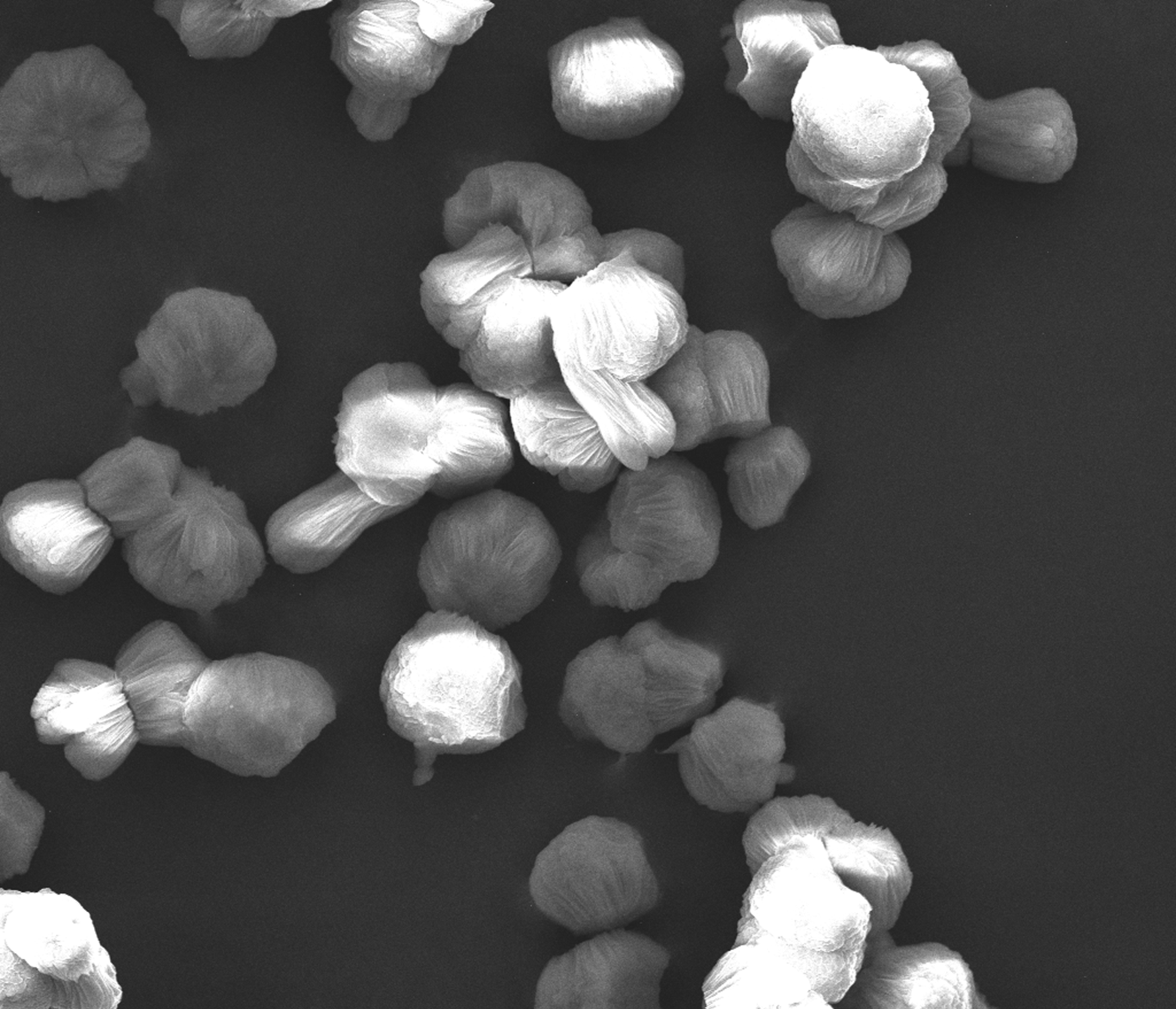


## Figure S14. SEM images of PCN-1005.


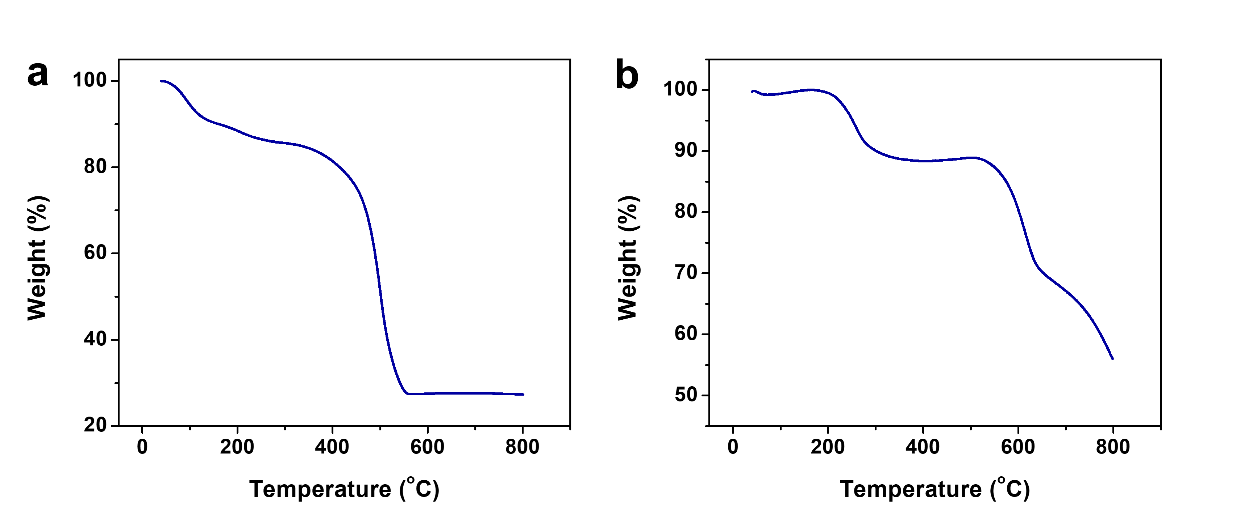


## Figure S15. TGA profiles of (a) PCN-1005 and (b) PCN-1006.

**
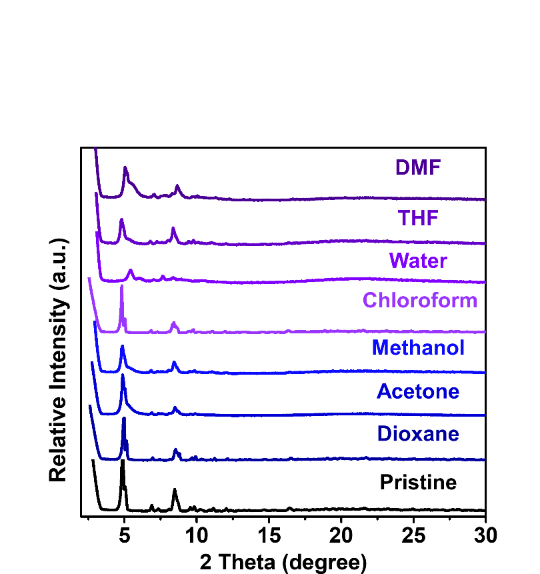
**

## Figure S16. PXRD patterns of PCN-1005 before and after exposure to various organic solvents.


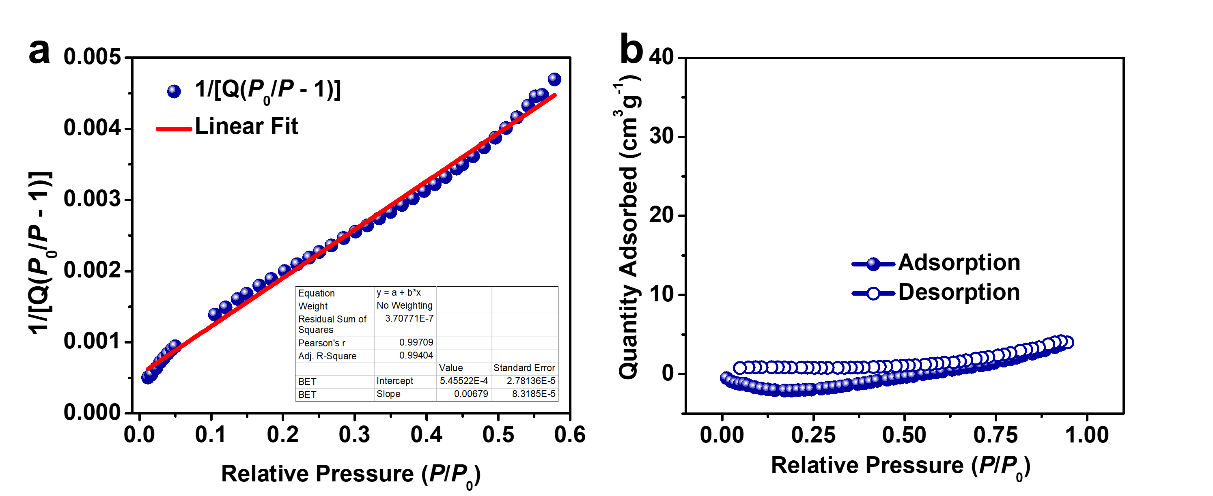


## Figure S17. (a) BET surface area plot of PCN-1005. (b) N_2_ adsorption-desorption isotherm of PCN-1005 at 77 K.


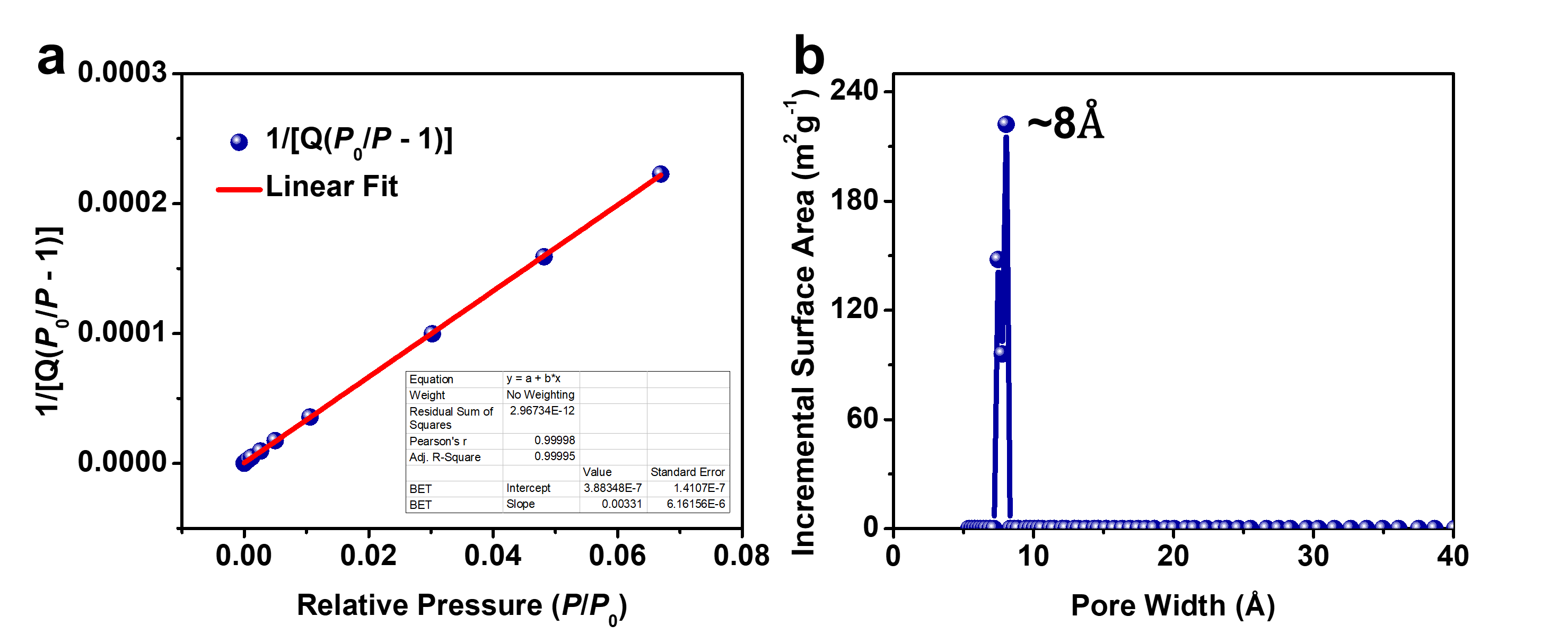


## Figure S18. (a) BET surface area plot of PCN-1006. (b) Pore size distribution profile of PCN-1006.


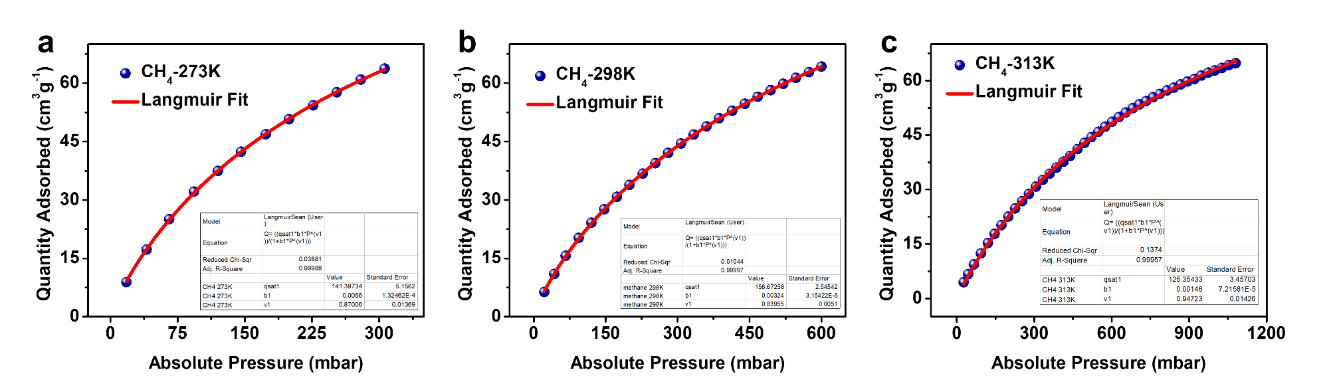


## Figure S19. Langmuir Fit of CH_4_ uptake of PCN-1006 at different temperatures.


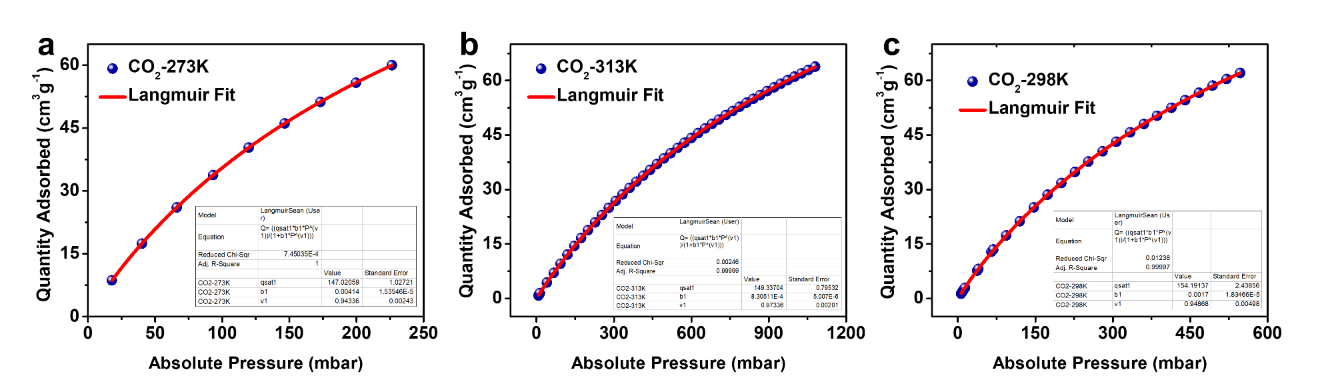


## Figure S20. Langmuir Fit of CO_2_ uptake of PCN-1006 at different temperatures.


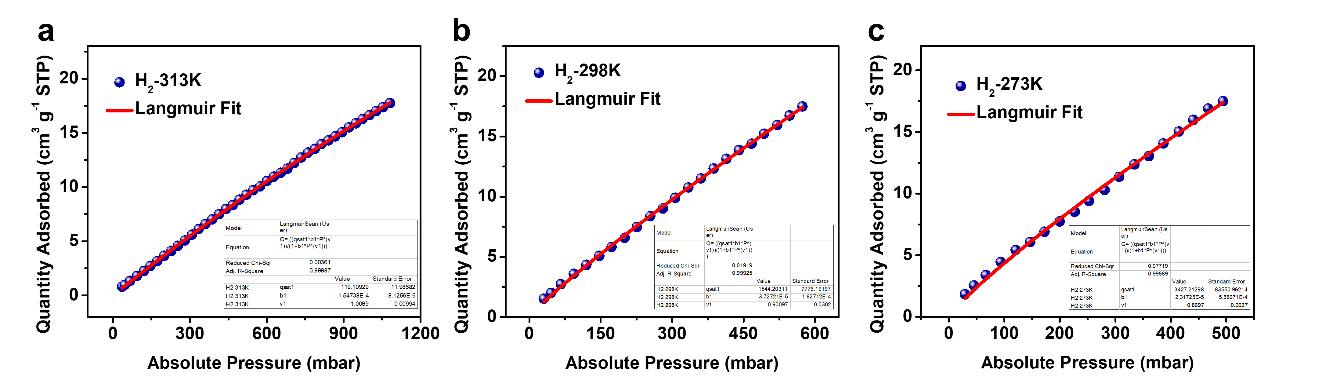


## Figure S21. Langmuir Fit of H_2_ uptake of PCN-1006 at different temperatures.


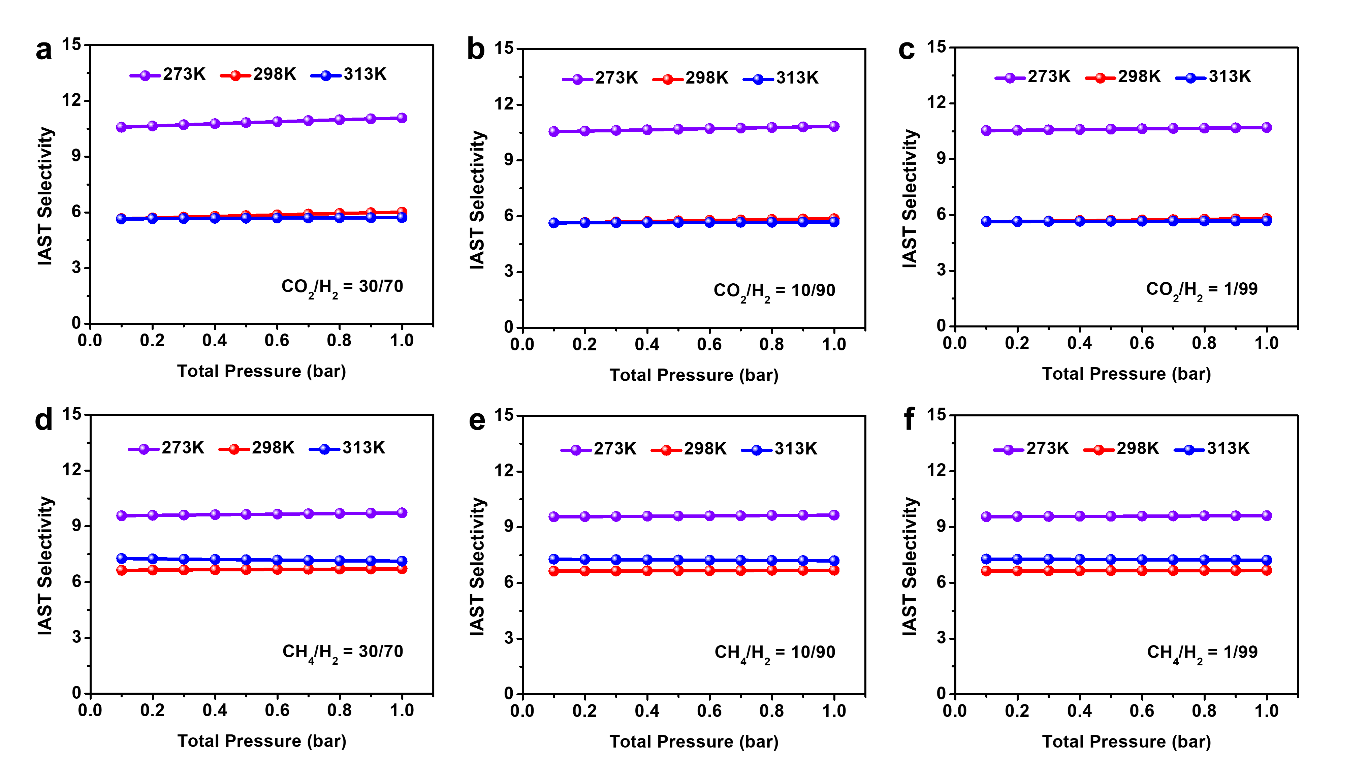


## Figure S22. IAST selectivity of (a) CO_2_/H_2_ (30/70), (b) CO_2_/H_2_ (10/90), (c) CO_2_/H_2_ (1/99), (d) CH_4_/H_2_ (30/70), (e) CH_4_/H_2_ (10/90), and (f) CH_4_/H_2_ (1/99).

## Figure S23. Breakthrough curves of two cycles for CO_2_/CH_4_/H_2_ (15/15/70) over PCN-1006 obtained at 298 K and 1 bar. Black: the first cycle; Red: the second cycle.

## Figure S24. PXRD patterns of PCN-1006 before and after breakthrough cycles.

## Table S1. Crystallographic data and structural refinement summary.

| Identification code | PCN-1005 | PCN-1006 |
| --- | --- | --- |
| CCDC | 2452803 | 2452802 |
| Empirical formula | C_104_H_81_NO_35_Zr_6_ | C_49_H_18_O_48_Zr_9_ |
| Formula weight | 2452.01 | 2195.61 |
| Temperature/K | 100.03(16) | 100 |
| Crystal system | orthorhombic | monoclinic |
| Space group | *P*2_1_2_1_2_1_ | *P*2_1_/*n* |
| *a*/Å | 34.4104(3) | 17.5286(15) |
| *b*/Å | 25.3123(2) | 27.933(2) |
| *c*/Å | 20.7123(2) | 22.6490(17) |
| *α*/° | 90 | 90 |
| *β*/° | 90 | 90.790(5) |
| *γ*/° | 90 | 90 |
| Volume/Å^3^ | 18040.5(3) | 11088.4(15) |
| *Z* | 4 | 4 |
| *ρ*_calc_ g/cm^3^ | 0.903 | 1.315 |
| *μ*/mm^‑1^ | 3.149 | 1.092 |
| *F*(000) | 4928.0 | 4224.0 |
| Crystal size/mm^3^ | 0.1 × 0.1 × 0.1 | 0.1 × 0.1 × 0.1 |
| Radiation | Cu Kα (λ = 1.54184) | synchrotron (λ = 0.7678) |
| 2*θ* range for data collection/° | 4.98 to 145.53 | 2.964 to 41.452 |
| Index ranges | -28 ≤ h ≤ 41, -30 ≤ k ≤ 30, -25 ≤ l ≤ 25 | -16 ≤ h ≤ 15, -25 ≤ k ≤ 25, -20 ≤ l ≤ 20 |
| Reflections collected | 88914 | 39230 |
| Independent reflections | 31748 [R_int_ = 0.0389, R_sigma_ = 0.0371] | 8829 [R_int_ = 0.0805, R_sigma_ = 0.0446] |
| Data/restraints/parameters | 31748/252/1267 | 8829/603/955 |
| Goodness-of-fit on F^2^ | 1.048 | 1.041 |
| Final R indexes [I>=2σ (I)] | R_1_ = 0.0546, wR_2_ = 0.1534 | R_1_ = 0.1450, wR_2_ = 0.3620 |
| Final R indexes [all data] | R_1_ = 0.0577, wR_2_ = 0.1578 | R_1_ = 0.1631, wR_2_ = 0.3799 |
| Largest diff. peak/hole / e Å^-3^ | 1.76/-1.79 | 1.94/-1.81 |

## Table S2. Summary of gas sorption and separation performance of reported adsorbents.

| **Materials** | **T**  **(K)** | **P**  **(bar)** | **CO_2_ Uptake**  **(mmol g^-1^)** | **CH_4_ Uptake**  **(mmol g^-1^)** | **H_2_ Uptake**  **(mmol g^-1^)** | **IAST Selectivity** | | **Ref.** |
| --- | --- | --- | --- | --- | --- | --- | --- | --- |
|  |  |  |  |  |  | **CO_2_/H_2_** | **CH_4_/H_2_** |  |
| activated carbon  (AX-21) | 298 | 1 | 2.3 | 1.4 | 0.20 | 23.21*^a^* | 10.35*^a^* | 3 |
| Mg-MOF-74 | 298 | 1 | 5.8 | 0.7 | 0.24 | 154.34*^a^* | 5.42*^a^* |  |
| Zn-MOF-74 | 298 | 1 | 4.3 | 0.5 | 0.16 | 94.58*^a^* | 5.92*^a^* |  |
| Co-MOF-74 | 298 | 1 | 4.7 | 0.4 | 0.17 | 136.13*^a^* | 4.37*^a^* |  |
| Ni-MOF-74 | 298 | 1 | 4.8 | 0.4 | 0.18 | 186.93*^a^* | 4.30*^a^* |  |
| Cu-MOF-74 | 298 | 1 | 3.1 | 0.2 | 0.07 | 107.01*^a^* | 5.49*^a^* |  |
| 3D-printed MOF-74 (Ni) | 298 | 10 | 4.0 | -- | 0.16 | 580*^b^* | -- | 4 |
| Zeolite-5A | 298 | 21 | 6.1 | 4.2 | 0.4 | 1000*^c^* | -- | 5 |
| MOF-74 | 298 | 21 | 10.6 | 6.6 | 0.6 | 1250*^c^* | -- |  |
| Zeo-A@MOF-74-1 | 298 | 21 | 13.8 | 7.7 | 0.9 | 1700*^c^* | -- |  |
| Mg_2_(dobdc) | 313 | 35 | 15 | 10 | 3 | 1650*^d^* | 12*^e^* | 6 |
| MOF-177 | 313 | 39 | 34 | -- | 3 | 7.5*^d^* | -- | 7 |
| BeBTB | 313 | 35 | 29 | -- | 6 | 7*^d^* | -- |  |
| CoBDP | 313 | 39 | 17 | -- | 3 | 2.5*^d^* | -- |  |
| CuBTTri | 313 | 39 | 17 | -- | 3 | 35*^d^* | -- |  |
| UiO-67(Zr)*^f^* | -- | 50 | 14 kmol m^-3^ | 9 kmol m^-3^ | 2 kmol m^-3^ | 50*^g^* | 15*^g^* | 8 |
| UiO-66(Zr)*^f^* | -- | 50 | 9 kmol m^-3^ | 7 kmol m^-3^ | 1.7 kmol m^-3^ | 200*^g^* | 65*^g^* |  |
| UiO-66(Zr)-Br*^f^* | -- | 50 | 8 kmol m^-3^ | 6 kmol m^-3^ | 1.5 kmol m^-3^ | 400*^g^* | 80*^g^* |  |
| Zr-Cl_2_Azo  BDC*^f^* | -- | 50 | 14 kmol m^-3^ | 8 kmol m^-3^ | 2 kmol m^-3^ | 25*^g^* | 10*^g^* |  |
| MOF-5 | 298 | 40 | 22 | 10 | 0.9 | 8*^e^* | 5*^e^* | 9 |
| PCN-1006 | 273 | 1 | 5.1 | 4.7 | 1.7 | 11.1*^g^* | 9.7*^g^* | This Work |
| PCN-1006 | 298 | 1 | 3.9 | 3.6 | 1.4 | 6.0*^g^* | 6.7*^g^* | This Work |
| PCN-1006 | 313 | 1 | 2.9 | 2.9 | 0.9 | 5.7*^g^* | 7.1*^g^* | This Work |

*^a^*The selectivity of CO_2_ (CH_4_)/H_2_ (35/65). *^b^*The selectivity of CO_2_/H_2_ (40/60). *^c^*The selectivity of CO_2_/H_2_ (15/85). *^d^*The selectivity of CO_2_/H_2_ (20/80). *^e^*The selectivity of CO_2_ (CH_4_)/H_2_ (50/50). *^f^*Using simulations. *^g^*The selectivity of CO_2_ (CH_4_)/H_2_ (30/70).

## Reference

[1] O. V. Dolomanov, L. J. Bourhis, R. J. Gildea, J. A. K. Howard, H. Puschmann, *J. Appl. Cryst.* **2009**, *42*, 339.

[2] G. M. Sheldrick, *Acta Crystallogr. A* **2008**, *64*, 112.

[3] X. Zhang, Q. Zheng, H. He, *Microporous Mesoporous Mater.* **2022**, *336*, 111899.

[4] S. Lawson, F. Rezaei, *ACS Sustainable Chem. Eng.* **2021**, *9*, 10902.

[5] Q. Al-Naddaf, A. A. Rownaghi, F. Rezaei, *Chem. Eng. J.* **2020**, *384*, 123251.

[6] Z. R. Herm, R. Krishna, J. R. Long, *Microporous Mesoporous Mater.* **2012**, *151*, 481.

[7] Z. R. Herm, J. A. Swisher, B. Smit, R. Krishna, J. R. Long, *J. Am. Chem. Soc.* **2011**, *133*, 5664.

[8] A.-M. Banu, D. Friedrich, S. Brandani, T. Düren, *Ind. Eng. Chem. Res.* **2013**, *52*, 9946.

[9] Q. Yang, C. Zhong, *J. Phys. Chem. B* **2006,** *110,* 17776.
